# Antisense Transcription in *Loci* Associated to Hereditary Neurodegenerative Diseases

**DOI:** 10.1007/s12035-018-1465-2

**Published:** 2019-01-04

**Authors:** Silvia Zucchelli, Stefania Fedele, Paolo Vatta, Raffaella Calligaris, Peter Heutink, Patrizia Rizzu, Masayoshi Itoh, Francesca Persichetti, Claudio Santoro, Hideya Kawaji, Timo Lassmann, Yoshihide Hayashizaki, Piero Carninci, Alistair R. R. Forrest, FANTOM Consortium, Stefano Gustincich

**Affiliations:** 1 Area of Neuroscience, SISSA, Trieste, Italy; 2 Department of Health Sciences and Interdisciplinary Research Center of Autoimmune Diseases (IRCAD), University of Piemonte Orientale (UPO), Novara, Italy; 3 Department of Neuroscience and Brain Technologies, Istituto Italiano di Tecnologia, Via Morego 30, 16163 Genoa, Italy; 4 Department of Medical, Surgical and Health Sciences, Clinical Neurology Unit, Cattinara University Hospital, Trieste, Italy; 5 Section Medical Genomics, Department of Clinical Genetics, VU University Medical Center, Amsterdam, The Netherlands; 6 Genome Biology of Neurodegenerative Diseases, Deutsches Zentrum fur Neurodegenerative Erkrankungen (DZNE), Standort, Tübingen, Germany; 7 Division of Genomic Technologies, RIKEN Center for Life Science Technologies, Yokohama, Japan; 8 RIKEN Omics Science Center, Yokohama, Japan; 9 Applied Genomics for Neurodegenerative Diseases, Deutsches Zentrum fur Neurodegenerative Erkrankungen (DZNE), Standort, Tübingen, Germany; 10 RIKEN Preventive Medicine and Diagnosis Innovation Program, Wakō, Japan; 11 Preventive Medicine and Applied Genomics Unit, RIKEN Center for Integrative Medical Sciences, Yokohama, Japan; 12 Telethon Kids Institute, The University of Western Australia, 100 Roberts Road, Subiaco, WA 6008 Australia; 13 Laboratory for Applied Computational Genomics, RIKEN Center for Integrative Medical Sciences, Yokohama, Japan; 14 Laboratory for Transcriptome Technology, RIKEN Center for Integrative Medical Sciences, Yokohama, Japan; 15 Laboratory for Genome Information Analysis, RIKEN Center for Integrative Medical Sciences, Yokohama, Japan

**Keywords:** Antisense transcription, Long noncoding RNA, Neurodegenerative diseases

## Abstract

Natural antisense transcripts are common features of mammalian genes providing additional regulatory layers of gene expression. A comprehensive description of antisense transcription in *loci* associated to familial neurodegenerative diseases may identify key players in gene regulation and provide tools for manipulating gene expression. We take advantage of the FANTOM5 sequencing datasets that represent the largest collection to date of genome-wide promoter usage in almost 2000 human samples. Transcription start sites (TSSs) are mapped at high resolution by the use of a modified protocol of cap analysis of gene expression (CAGE) for high-throughput single molecule next-generation sequencing with Helicos (hCAGE). Here we present the analysis of antisense transcription at 17 *loci* associated to hereditary Alzheimer’s disease, Frontotemporal Dementia, Parkinson’s disease, Amyotrophic Lateral Sclerosis, and Huntington’s disease. We focused our analysis on libraries derived from brain tissues and primary cells. We also screened libraries from total blood and blood cell populations in the quest for peripheral biomarkers of neurodegenerative diseases. We identified 63 robust promoters in antisense orientation to genes associated to familial neurodegeneration. When applying a less stringent cutoff, this number increases to over 400. A subset of these promoters represents alternative TSSs for 24 FANTOM5 annotated long noncoding RNA (lncRNA) genes, in antisense orientation to 13 of the *loci* analyzed here, while the remaining contribute to the expression of additional transcript variants. Intersection with GWAS studies, sample ontology, and dynamic expression reveals association to specific genetic traits as well as cell and tissue types, not limited to neurodegenerative diseases. Antisense transcription was validated for a subset of genes, including those encoding for Microtubule-Associated Protein Tau, α-synuclein, Parkinsonism-associated deglycase DJ-1, and Leucin-Rich Repeat Kinase 2. This work provides evidence for the existence of additional regulatory mechanisms of the expression of neurodegenerative disease-causing genes by previously not-annotated and/or not-validated antisense long noncoding RNAs.

**Electronic supplementary material:**

The online version of this article (10.1007/s12035-018-1465-2) contains supplementary material, which is available to authorized users.

## Background

Natural antisense (AS) transcripts are RNA molecules that are transcribed from the opposite DNA strand to sense (S) transcripts, partially or fully overlapping to form S/AS pairs. It is now well documented that AS transcription is a common feature of genomes from bacteria to mammals [1]. S/AS pairs are estimated to cover more than 70% of the whole transcriptome in humans [2, 3], thus suggesting a crucial role in gene expression control. S/AS can overlap at their 5′ end forming a head-to-head divergent pair or at their 3′ establishing a tail-to-tail convergent one. They are fully overlapping when the extremities of one gene are contained within the other one. S/AS pairs can present all the combinations between protein-coding and long noncoding RNAs (lncRNA), while the most studied configuration presents a protein-coding gene together with a lncRNA on the opposite strand [1].

LncRNAs are defined as transcripts longer than 200 nucleotides, with features similar to that of protein-coding genes but without a functional open reading frame (ORF). Thousands of lncRNA genes have been identified in mammalian genomes, with their number increasing steadily [4–8]. It is now clear that lncRNAs can regulate several biological processes, including those that underlie human diseases and yet their detailed functional characterization remains limited. As representative examples, in the nucleus, lncRNAs can have enhancer-like activity [9], they contribute to control the epigenetic status of the chromatin through recruitment of chromatin-modifying complexes [10–13], they are required for the formation of subnuclear structures [14, 15] and higher order chromatin assemblies [16], and they can act as decoy for inhibiting splicing and mRNA maturation [17]. In the cytoplasm, lncRNAs were described to activate mRNA decay via Alu elements [18], to act as “sponges” for miRNA binding [19, 20] or even to modulate cytoplasmic protein enzymatic activity [21]. Importantly, lncRNAs present unprecedented opportunities to modify gene expression at the right time in the correct space in vivo, providing an almost unlimited reservoir of new potential pharmacological agents [22].

When part of S/AS pairs, antisense lncRNAs control the output of the protein encoding transcriptome acting at distinct regulatory levels. They can modulate methylation of DNA and/or histones [23], they can promote sense gene transcription by recruiting transcription factors to enhancers or modulate splicing of sense pre-mRNA [17, 24–28], and they can control the half-life of their sense partners by establishing Dicer-dependent cutting of dsRNA, potentially followed by siRNA-mediated gene silencing [29]. In the cytoplasm, they can promote translation of the overlapping sense mRNA [30]. Antisense transcripts can also contribute to genetic diseases since a mutation in the antisense gene led to *HBA2* gene silencing and DNA methylation in a α-thalassemia patient [31].

The current understanding of the role of antisense lncRNAs to brain function and neurodegenerative diseases remains scarce. An antisense transcript across expanded CGG repeats in the 5′ UTR of the fragile X mental retardation gene has been found upregulated in individuals with permutation alleles and therefore implicated in the pathogenesis of fragile X syndrome and fragile X-associated tremor and ataxia syndrome [32]. A lncRNA antisense to *BACE1* (*BACE1-AS*) plays a prominent role in regulating *BACE1* mRNA levels, thus contributing to Aβ-induced toxicity in Alzheimer’s disease (AD) [33]. Its mechanism of action involves competition for microRNA-binding sites [34]. Expression of specific natural antisense transcripts in the region of CAG/polyglutamine repeats affects transcription of the corresponding sense gene in Huntington’s disease (HD) and spinocerebellar ataxia type 7 [35, 36]. A noncoding antisense transcript at the *PINK1* locus is positively co-regulated with a splice variant of *PINK1* mRNA in human cell lines [37]. In 2011, two independent studies demonstrated that the most common cause of familial forms of Frontotemporal Dementia (FTD) and/or Amyotrophic Lateral Sclerosis (ALS) is the expansion of hexanucleotide repeats within the *C9orf72* gene [38, 39]. Unexpectedly, antisense transcription of the expanded repeats contributes to *C9orf72* neurotoxicity giving rise to non-AUG translated aggregating repeat peptides and to antisense RNA foci in the brain of disease patients [40, 41]. Fine mapping of *C9orf72* expression in the brain and blood of patients and individuals without repeat expansion identified three distinct antisense transcripts, namely *C9orf72-AS1*, *AS2*, and *AS3*, that are highly expressed in myeloid cells and less in the brain and that may contribute to gene regulation in a cell-type-specific manner [42]. Genes associated to familial neurodegenerative diseases may also present antisense protein-coding transcripts. *PACRG* was found highly co-regulated with its sense *PRKN* mRNA [43]. Interestingly, *KIAA1267*, antisense to *MAPT*, and *DDOST*, antisense to *PINK1*, were found mutated together with their sense gene in disease.

A major limit in our current understanding on the role of antisense transcription to neurodegenerative diseases derives from the lack of a comprehensive approach. Studies are usually restricted to a single gene of interest at a time, and validation experiments are carried out only in selected brain area/cell type. Cap analysis of gene expression (CAGE) is a technology based on the generation of short sequence tags from the 5′ end of full-length cDNAs followed by high-throughput sequencing. When mapped to a reference genome, CAGE tags survey transcription start site (TSS) activity of specific promoters and measure expression levels on a massive scale [44, 45]. The FANTOM5 project has developed a simplified CAGE protocol adapted to the single-molecule HeliScope sequencer (hCAGE) [46] to decrease PCR biases and improve depth of sequencing. hCAGE allows to quantify TSSs at single base pair resolution from small amounts of starting material. hCAGE technology was used to profile almost 2000 human and 1000 mouse samples to build a promoter-level mammalian expression atlas and to model networks of distinct cellular states [47–49]. Recently, the FANTOM5 Consortium has integrated hCAGE data with multiple transcript collections to generate a comprehensive atlas of 27,919 human lncRNA genes [6]. The peculiarity of the FANTOM5 lncRNA catalog is the accurate definition of the 5′ end for each transcript and its expression profiling in almost 2000 human libraries. The FANTOM5 assembly has been referred to as FANTOM CAGE-Associated Transcriptome or FANTOM CAT [6]. In addition to gene annotation, FANTOM CAT presents genomic and epigenomic classification of lncRNA transcripts and intersection with genetic data thus providing cues of their functional relevance.

To help better understand the impact of complex gene regulatory networks to neurodegeneration, as part of the FANTOM5 Consortium, we surveyed the antisense transcriptional landscape in chromosomal regions of 17 genes associated to familial forms of neurodegenerative diseases, including AD, FTD, PD, ALS, and HD. We focused our attention on brain-derived samples and on blood libraries for the prominent role of this tissue in peripheral biomarker discovery. We complemented this work mining the recently compiled catalog of human lncRNAs to intersect expression with genomic annotation and association to disease traits. Our analysis reveals extensive expression of multiple TSSs in antisense orientation to sense neurodegenerative disease-associated genes, thus identifying novel potential players in neurodegeneration. In selected cases, epigenetic marks imply their possible contribution as promoter-associated or enhancer-associated antisense lncRNAs. Most importantly, association studies point toward an antisense lncRNA-based pleiotropy between neurodegenerative diseases and pathologies outside the nervous system.

This work is part of the FANTOM5 project. Data downloads, genomic tools, and co-published manuscripts are summarized here (http://fantom.gsc.riken.jp/5/).

## Methods

### FANTOM5 Data Analysis

Analysis of FANTOM5 collection of human libraries was performed using Zenbu browser genomic data visualization tool (http://fantom.gsc.riken.jp/5/). A specific script was designed to extract expression values from graphical tables in Zenbu genome browser and convert into Excel spreadsheet for further analysis (Zenbu utility tool, unpublished). FANTOM CAT resource in Zenbu (http://fantom.gsc.riken.jp/cat/) was used to retrieve data on genome-wide association studies (GWAS), sample ontology enrichment, genomic features, assembly, and classification of human lncRNAs in the newly defined FANTOM5 catalog. For sample ontology, we used both UBERON (tissues) and CL (cells) terms. A set of nonredundant sample ontology terms [49] was selected on the basis of manual curation to describe a subset of FANTOM5 cell and tissue samples. Each curated sample ontology term and its association to the selected samples are included, as supplementary table, in a previously published work [6]. Enrichment in a specific ontology term is based on gene expression in samples falling in the same sample ontology term.

Trait-associated SNPs were derived by publicly available databases (as of June 2015): (1) GWASdb, for genome-wide association SNPs, and (2) probabilistic identification of causal SNPs (PICS), for fine-mapped SNPs.

Venn diagrams were prepared using bioinformatics online tool (http://bioinformatics.psb.ugent.be/webtools/Venn/). Heatmaps were produced with the heatmap2 R package with default parameters or using web-enabled heatmapper (http://www1.heatmapper.ca/expression/) [50].

### Human Blood Samples and RNA Isolation

Blood sample was obtained from the Neurologic Clinic of Trieste (Italy) from a healthy volunteer upon written informed consent and the institutional approval of the Ethical Committee at the Movement Diseases Center. The subject was a Caucasian young adult and showed absence of neurological symptoms and of metabolic and vascular diseases. Venous peripheral blood was collected directly into PAXgene Blood RNA tubes (PreAnalytiX) via a 21-gauge butterfly needle and immediately frozen. Total RNA was extracted using PAXgeneTM Blood RNA kit (PreAnlytiX) according to the manufacturer’s instructions. RNA quality was evaluated on a 2100 Bioanalyzer (Agilent Technologies, Santa Clara, CA), and RNA quantity was measured using a NanoDrop spectrophotometer (NanoDrop Technologies). The RNA was stored at − 80 °C until further analysis.

### Human SH-SY5Y Neuroblastoma Cell Culture and RNA Isolation

Human neuroblastoma cell line SH-SY5Y was obtained from ATCC (CRL-2266™) and maintained in culture in 1:1 E-MEM:F-12 media supplemented with 15% fetal bovine serum, 2 mM glutamine, 1% nonessential amino acids, 100 μg/ml penicillin, and 100 μg/ml streptomycin (Invitrogen).

Total RNA was extracted from subconfluent SH-SY5Y cells using TRIzol reagent (Invitrogen) and following the manufacturer’s instructions.

### Reverse Transcription and PCR

RNA samples isolated from human SH-SY5Y cells and human whole blood were treated with DNAse I (Ambion) before use. A panel of purified and DNAse-treated human tissue-specific RNAs was obtained from a commercial source (FirstChoice® Human Total RNA Survey Panel, Life Sciences). The panel consists of 20 different normal human tissues. In each tissue sample, RNA is derived by at least three donors with full documentation on age, sex, race, and cause of death.

Single-strand cDNA was prepared from 1 μg of purified RNA using the iSCRIPT™ cDNA Synthesis Kit (Bio-Rad) according to the manufacturer’s instructions. Nonquantitative PCR was performed with standard protocol. Quantitative real-time PCR (qRT-PCR) was performed using SYBR-Green PCR Master Mix (Applied Biosystems) and an iCycler IQ Real-time PCR System (Bio-Rad). Oligonucleotide sequences of primers used in this study are indicated below. The amplified transcripts were quantified using the comparative Ct method, and the differences in gene expression were presented as normalized fold expression (ΔΔCt). Expression in the “brain” was set to 1. All of the experiments were performed in duplicate. A heatmap graphical representation of normalized fold expression was obtained by using a web-enabled heatmapper [50].

Amplicons obtained by nonquantitative and quantitative PCRs were subcloned and subjected to Sanger sequencing using custom services (IGA, Udine, Italy, and Eurofins-MGW Operon, Germany).

### Primers for Nonquantitative PCR

The following oligonucleotides were used for nonquantitative PCR of AS transcripts. When primers are designed on an annotated transcript, the accession number is indicated and primers are designed intron-spanning. Primers to amplify nonannotated AS transcripts are positioned around TSS sequence and across exon–intron boundaries of sense mRNA.

- AS APP (E455275) fwd GAAGGCTCAGCTCTTGGATG
- AS APP (E455275) rev TCCTTCTGTCTTGGAGGAGGT
- AS APP (E456904) fwd CATGCGGTTTGTGGTAACTG
- AS APP (E456904) rev CCTTCCACTTCCTTGCTGAG
- DJ1-5′AS fwd AGGGTGGCGGTAGAGACTGT
- DJ1-5′AS rev CACACCAGGCTGAAAATGAA
- LRRK2-5′AS (set 1) fwd GTGGAATCTGGTCCCAGGAG
- LRRK2-5′AS (set 1) rev GGCTGGGATGACCGTGAC
- LRRK2-5′AS (set 2) fwd ACGGTCACGGTCATCCCA
- LRRK2-5′AS (set 2) rev CCCAAAAACGAGCTCAGG
- LRRK2-5′AS (set 3) fwd GCCCCTGAGCTCGTTTTT
- LRRK2-5′AS (set 3) rev CATGAACGTCCGCTGCTC
- AS SNCA (set 1) fwd (E513653) CTCCAGGATTTCCAAAGACG
- AS SNCA (set 1) rev (E513653) CGTGTATTTCTTGGGCTCATT
- AS SNCA (set 2) fwd (E501215) CTCCAGGATTTCCAAAGACG
- AS SNCA (set 2) rev (E501215) CGCAAGAATCAGACAAAGCA
- SNCA-5′AS (set 3) fwd GCTCCTCGTCCCTATCTCG
- SNCA-5′AS (set 3) rev CCGATCTCCACAAGAGTGCT
- SNCA-5′AS (set 4) fwd TGAGGGTCGACCACCAAG
- SNCA-5′AS (set 4) rev AGCGACCCCTAACGTTGTAA
- SNCA-intAS (set 5) fwd CCCATCACTCATGAACAAGC
- SNCA-intAS (set 5) rev GCAGAAGCAGCAGGAAAGAC

Oligonucleotides to beta-actin transcripts were included as controls:

- Actin fwd CGCCGCCAGCTCACCATG
- Actin rev CACGATGGAGGGGAAGACGG

### Primers for Quantitative RT-PCR

The following oligonucleotides were used for quantitative real-time PCR. All primers of sense genes were intron-spanning.

#### Sense genes

- DJ-1-sense fwd GGAGACGGTCATCCCTGTAG
- DJ-1-sense rev GGCTACACTGTACTGGGTCTTT
- LRRK2-sense fwd ATGTGCCTCTGTTGATCGTCT
- LRRK2-sense rev TTGCACAGAAGTGACCAACC
- MAPT-sense fwd GCAAGGTGACCTCCAAGTGTGGCT
- MAPT-sense rev TCCTCCGCCAGGGACGTGGG
- SNCA-sense fwd TCTCTATGTAGGCTCCAAAACCAA
- SNCA-sense rev TGCTCCTCCAACATTTGTCAC
- Actin fwd/rev

#### Antisense transcripts

- DJ1-5′AS fwd/rev same as for nonquantitative PCR
- LRRK2-5′AS (set 1) fwd/rev same as for nonquantitative PCR
- SNCA-intAS (set 5) fwd/rev same as for nonquantitative PCR

## Results

### Selection of Neurodegenerative Disease-Associated Genes

For our analysis, we selected a set of genes that have been demonstrated to cause familial forms of AD, FTD, PD, ALS, and HD. We interrogated the NCBI OMIM database and disease-specific databases (PDmut Database, AD/FTDmut Database, ALSmut Database) to focus on monogenic forms. Genes were crosschecked with the most recent literature [51–55]. The genes enrolled in our analysis include amyloid beta precursor protein (*APP*), presenilin1 (*PSEN1*) and presenilin2 (*PSEN2*) for AD; chromosome 9 open reading frame 72 (*C9orf72*), microtubule-associated protein tau (*MAPT*), and granulin precursor (*GRN*) for FTD; α-synuclein (*SNCA*), parkin RBR E3 ubiquitin protein ligase (*PRKN*), PTEN-induced putative kinase 1 (*PINK1*), Parkinsonism-associated deglycase DJ-1 (*PARK7*), leucine-rich repeat kinase 2 (*LRRK2*), and VPS35 retromer complex component (*VPS35*) for PD; superoxide dismutase1 (*SOD1*), FUS RNA-binding protein (*FUS*), TAR DNA-binding protein (*TARDBP*), and ubiquilin2 (*UBQLN2*) for ALS and Huntingtin (*HTT*) for HD (Table 1). We are aware that the list of genes selected in our study does not encompass all monogenic cases associated to the most common neurodegenerative disorders. A number of genetic studies, for instance, have unveiled the role of *VPS13C* and *ATP13A2* in PD [56–58] or *VCP* and *CHMP2B* in FTD [54]. Moreover, a complex pattern of genetic and clinical overlaps exists in patients. This is particularly true for FTD and ALS, as demonstrated by *C9orf72* cases. Due to space limitations and to help readers through the data, we have selected the most common neurodegenerative diseases and we have focused our attention on three to four biologically validated genes for each disease, establishing a simplified “gene by disease” classification.

**Table 1:** List of neurodegenerative disease-associated genes recruited for this study. Gene name, gene symbol, disease symbol, and inheritance are shown

Gene name	Gene symbol	Disease	Inheritance
Amyloid beta precursor protein	*APP*	AD	Autosomal dominant
Presenilin 1	*PSEN1*	AD	Autosomal dominant
Presenilin 2	*PSEN2*	AD	Autosomal dominant
Chromosome 9 open reading frame 72	*C9orf72*	FTD	Autosomal dominant
Granulin precursor	*GRN*	FTD	Autosomal dominant
Microtubule-associated protein tau	*MAPT*	FTD	Autosomal dominant
Synuclein alpha	*SNCA*	PD	Autosomal dominant
Parkin RBR E3 ubiquitin protein ligase	*PRKN*	PD	Autosomal recessive
PTEN-induced putative kinase 1	*PINK1*	PD	Autosomal recessive
Parkinsonism associated deglycase (DJ-1)	*PARK7*	PD	Autosomal recessive
Leucine-rich repeat kinase 2	*LRRK2*	PD	Autosomal dominant
VPS35, retromer complex component	*VPS35*	PD	Autosomal dominant
FUS RNA-binding protein	*FUS*	ALS	Autosomal dominant
Superoxide dismutase 1	*SOD1*	ALS	Autosomal dominant
TAR DNA-binding protein	*TARDBP*	ALS	Autosomal dominant
Ubiquilin 2	*UBQLN2*	ALS	Autosomal dominant
Huntingtin	*HTT*	HD	Autosomal dominant

*AD*, Alzheimer’s disease; *FTD*, frontotemporal dementia; *PD*, Parkinson’s disease; *ALS*, amyotrophic lateral sclerosis; *HD*, Huntington’s disease

### Antisense Transcription at Neurodegenerative Disease-Associated *Loci*

To characterize the antisense transcriptional landscape occurring at the genomic *loci* that we have selected and to identify new TSSs that may contribute to gene regulation, we used hCAGE expression data generated by the FANTOM5 Consortium.

A decomposition-based peak identification method (DPI) was used to identify hCAGE peaks across the genome and thus annotate promoters [49]. Each DPI cluster, composed by multiple hCAGE signals, represents a TSS. To minimize for peaks mapping to internal exons and to enrich for real TSS, FANTOM5 applied tag evidence thresholds to define robust DPI and permissive DPI sets. For our analysis, we decided to focus on bona fide TSSs, derived from robust DPI cluster and with an expression cutoff of at least three tags/library. When necessary, we also mined the permissive set, to give a larger glimpse at the genomic architecture of each locus. Zenbu (http://fantom.gsc.riken.jp/5/), a web-based and open-source genome browser developed by the Consortium, was used for interactive data exploration and visualization [59, 60].

Antisense TSSs are present for the majority of neurodegenerative disease-associated *loci* that we have investigated, with 12 genes out of 17 having at least one robust antisense DPI cluster (Table 2). Since our main interest is to dissect the genomic complexity and reveal possible impact of antisense lncRNAs to neurodegeneration, we excluded from our counting those antisense TSSs derived by protein coding already described at these *loci*, albeit relevant to the pathology. This is the case for Parkin co-regulated gene (*PACRG*) antisense to *PRKN*, *FAM171A2* antisense to *GRN*, *KIAA1267* to *MAPT*, and *DDOST* in the locus of *PINK1*. Using our stringent cutoff, we counted a total of 63 TSSs, with an average of 3.7 TSS/gene spanning from a minimum of 1 to a maximum of 12. The quantity of antisense TSSs only partially correlates with gene size. While larger genes (*PRKN* and *APP*) incorporate 12 antisense TSSs each across the entire locus, genes with similar size (180–120 kb) have zero (*HTT*) or up to 10 antisense promoters (*SNCA*). It has to be noted that when including the permissive set of DPI clusters, a much more complex antisense transcription architecture is observed at these *loci*. Indeed, a total of 403 permissive TSSs exist, covering every single gene in the analysis. *MAPT*, followed by *PRKN* and *APP*, has the highest level of antisense signals (74 TSSs), while PSEN2 has the least (1 TSS) (Supplementary Table S1).

**Table 2:** Catalog of FANTOM5 promoters in *loci* associated to hereditary neurodegenerative diseases. For each gene (gene symbol), the total number of robust TSSs (TSS DPI robust) and CAT clusters (CAT CAGE cluster robust) is indicated. The total number of promoters identified in this study is also shown (at the bottom)

Disease	Gene symbol	TSS DPI robust	CAT CAGE cluster robust
AD	*APP*	12	5
AD	*PSEN1*	5	2
AD	*PSEN2*	1	0
FTD	*C9orf72*	4	1
FTD	*GRN*	0	0
FTD	*MAPT*	8	6
PD	*SNCA*	10	8
PD	*PRKN*	12	6
PD	*PINK1*	2	6
PD	*PARK7*	2	0
PD	*LRRK2*	4	4
PD	*VPS35*	0	1
ALS	*SOD1*	2	6
ALS	*FUS*	0	6
ALS	*TARDBP*	0	2
ALS	*UBQLN2*	1	2
HD	*HTT*	0	1
	Total	63	56

The expression level, measured as tag per million (TPM) across each locus, is always lower in antisense orientation as compared to the reference gene in direct orientation. In particular, the majority of transcriptional signal is concentrated in the main sense TSS, thus identifying the main promoter for each protein-coding gene. Values for sense and antisense transcription are extremely variable across libraries, thus indicating tissue- and cell-specific regulation of antisense promoter activity.

### Genomic Features and Categories of Antisense LncRNAs at Neurodegenerative Disease-Associated *Loci*

To gain insights into the significance of the antisense TSSs we have identified, we used FANTOM CAT data visualization in Zenbu (http://fantom.gsc.riken.jp/cat/) and mined CAT assembly of human lncRNAs. To expand the coverage of lowly abundant transcripts, in FANTOM CAT unfiltered and nonoverlapping clusters derived from time-course analysis were also added to the robust CAGE clusters identified with the DPI method and described in the original promoter atlas. The resulting TSSs were defined as CAT CAGE clusters [6]. We found 56 CAGE clusters annotated in FANTOM CAT (Table 2). The relative distribution of DPI and CAT CAGE clusters in each locus shows some differences. About half of the neurodegenerative disease-associated genes, namely *APP*, *PSEN1*, and *PSEN2* for AD; *C9orf72* and *MAPT* for FTD and *SNCA*; and *PRKN* and *PARK7* for PD, have larger number of DPI robust TSSs than annotated CAT CAGE clusters. This suggests the existence of novel TSSs that are not included in any transcript assembly model available so far. For the remaining genes, *PINK1* and *VPS35* for PD and all the genes associated to ALS, we found more CAT clusters than robust TSSs. This is explained by the stringent cutoff parameters applied to the robust DPI set, as the above genes have large numbers of antisense TSSs included in the permissive DPI set (Supplementary Table S1).

**Fig. 1:**
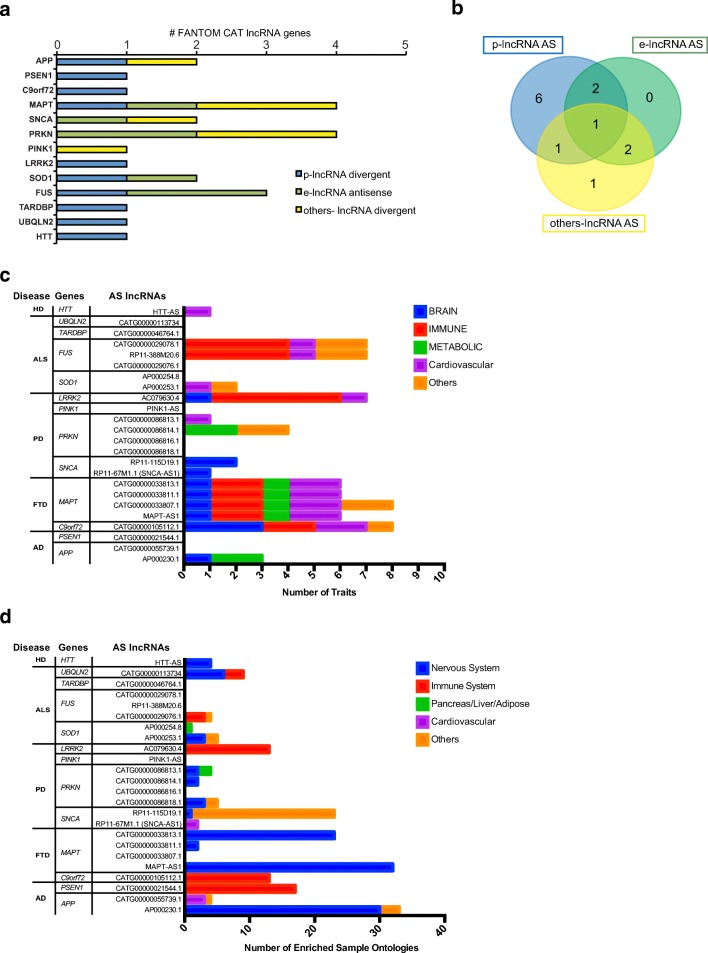
FANTOM CAT annotation of antisense lncRNAs at *loci* of genes associated to familial forms of neurodegenerative diseases. **a** Numbers and categories of antisense lncRNAs based on FANTOM CAT annotation: in blue, promoter-associated lncRNAs (p-lncRNAs); in green, enhancer lncRNAs (e-lncRNAs); in yellow, unidentified classification of lncRNAs (others-lncRNAs). Gene symbols are shown on the *y*-axis. Numbers of FANTOM CAT antisense lncRNAs are indicated on the *x*-axis. **b** Distribution of FANTOM CAT categories of lncRNAs (color coding as in **a** across *loci* associated to neurodegenerative diseases. **c** Enrichment of GWAS and PICS SNPs at lncRNA genes that are located antisense to selected neurodegenerative diseases-associated genes using FANTOM CAT. Disease types are grouped in the indicated categories (brain, immune, metabolic, cardiovascular, and others). Number of disease traits is plot for each antisense lncRNA gene (*x*-axis at the bottom). Relative protein-coding gene (Gene) and associated causative neurodegenerative disease (Disease) are shown (*y*-axis). **d** Analysis of sample ontology enrichment using FANTOM CAT. Number of enriched sample ontologies is shown for each antisense lncRNA gene. Causative gene and neurodegenerative disease type are also indicated. Ontology types are grouped in the indicated categories (Nervous System, Immune System, Pancreas/Liver/Adipose Tissues, Cardiovascular, Others)

Overlay of CAT clusters with gene annotation identified 32 antisense genes for 14 of the neurodegeneration-associated *loci*, for which multiple TSSs are present (Table 3). Of these 32 antisense genes, two show putative coding potentials, while six are annotated as short noncoding RNA genes. Interestingly, 24 of 32 CAT assemblies, targeting 13 neurodegeneration *loci*, are classified as bona fide antisense lncRNA genes. Ten of these are located at the 5′ of the protein-coding gene, giving rise to a 5′ head-to-head divergent sense/antisense pair. Genomic categorization indicates that they belong to the class of promoter-associated lncRNAs (p-lncRNAs). Another seven antisense genes show epigenetic features of enhancer-associated lncRNA genes (e-lncRNAs), while no specific categories are indicated for the remaining seven lncRNAs. Distribution of annotated antisense lncRNA genes across the 13 genes indicates that the majority of them are under the control of a promoter-associated lncRNA (10 out of 13) (Fig. 1a, b). e-lncRNAs are associated to *APP*, *MAPT*, *PRKN*, and *PINK1*. In general, *MAPT* and *PRKN* appear to display the more complex pattern of antisense transcription.

**Table 3:** List of FANTOM CAT annotated antisense lncRNA genes identified in *loci* associated to hereditary neurodegenerative diseases. FANTOM CAT nomenclature (AS lncRNA gene) and annotation (AS lncRNA category) are indicated. TSS nomenclature used in this study is also included (AS TSS nomenclature)

Disease	Gene name	FANTOM CAT		This work
		AS lncRNA gene	AS lncRNA category	AS TSS nomenclature
AD	*APP*	*AP000230.1*	p-lncRNA divergent	APP-5′AS
AD	*APP*	*CATG00000055743.1*	Others-short ncRNA	APP-int2AS
AD	*APP*	*CATG00000055739.1*	Others- lncRNA divergent	APP-int3AS
AD	*APP*	*CATG00000055737.1*	Others-short ncRNA	APP-3′AS
AD	*PSEN1*	*CATG00000021544.1*	p-lncRNA divergent	PSEN1-5′AS
AD	*PSEN1*	*CATG00000021545.1*	Others-short ncRNA	PSEN1-intAS
FTD	*C9orf72*	*CATG00000105112.1*	p-lncRNA divergent	C9orf-5′AS (AS3)*
FTD	*C9orf72*	*CATG00000105110.1*	Others-uncertain coding	C9orf-3′AS
FTD	*MAPT*	*MAPT-AS1*	p-lncRNA divergent	MAPT-5′AS
FTD	*MAPT*	*CATG00000033807.1*	Others-lncRNA antisense	MAPT-int1AS
FTD	*MAPT*	*CATG00000033811.1*	Others-lncRNA antisense	MAPT-int2AS
FTD	*MAPT*	*CATG00000033813.1*	e-lncRNA antisense	MAPT-int3AS
PD	*SNCA*	*RP11-67 M1.1 (SNCA-AS1)*	Others-lncRNA divergent	SNCA-int1AS
PD	*SNCA*	*CATG00000069387.1*	Others-short ncRNA	SNCA-int2AS
PD	*SNCA*	*RP11-115D19.1*	e-lncRNA antisense	SNCA-3′AS2
PD	*PRKN*	*CATG00000086818.1*	e-lncRNA antisense	PRKN-int1AS
PD	*PRKN*	*CATG00000086816.1*	Others-lncRNA antisense	PRKN-int2AS
PD	*PRKN*	*CATG00000086814.1*	Others-lncRNA antisense	PRKN-int3AS
PD	*PRKN*	*CATG00000086813.1*	e-lncRNA antisense	PRKN-int4AS
PD	*PRKN*	*CATG00000086812.1*	Others-uncertain coding	PRKN-int5AS
PD	*PINK1*	*PINK1-AS*	Others-lncRNA antisense	PINK1-AS*
PD	*LRRK2*	*AC079630.4*	p-lncRNA divergent	LRRK2-5′AS
PD	*VPS35*	*CATG00000027205.1*	Others-short ncRNA	VPS35-AS
ALS	*SOD1*	*AP000253.1*	p-lncRNA divergent	SOD1-5′AS
ALS	*SOD1*	*AP000254.8*	e-lncRNA antisense	SOD1-int2AS
ALS	*FUS*	*CATG00000029076.1*	p-lncRNA divergent	FUS-5′AS
ALS	*FUS*	*RP11-388M20.6*	e-lncRNA divergent	FUS-int1AS
ALS	*FUS*	*CATG00000029078.1*	e-lncRNA divergent	FUS-int2AS
ALS	*TARDBP*	*CATG00000046764.1*	p-lncRNA divergent	TARDBP-AS
ALS	*UBQLN2*	*CATG00000113734*	p-lncRNA divergent	UBQLN2-int1AS
ALS	*UBQLN2*	*CATG00000113735*	Others-short ncRNA	UBQLN2-int2AS
HD	*HTT*	*HTT-AS*	p-lncRNA divergent	HTT-AS*

*AS lncRNAs in the literature

### Genetic Pleiotropy of Neurodegenerative Disease-Associated Antisense LncRNAs

It has been calculated that more than 85% of disease-related single nucleotide polymorphisms (SNPs) are within noncoding regions of the genome and are surprisingly overrepresented at enhancers and promoters, where antisense transcription also occurs. These data imply that SNPs mapping to promoter-associated and enhancer-associated lncRNAs may have a previously underestimated impact on human diseases. Since genetic pleiotropy is under intense scrutiny for neurodegenerative diseases, we checked the enrichment of GWAS and PICS SNPs at lncRNA genes that are located antisense to our neurodegenerative disease-associated genes using FANTOM CAT. We found that most FANTOM CAT annotated antisense lncRNAs to *APP*, *C9orf72*, *MAPT*, *SNCA*, *LRRK2*, *SOD1*, *FUS*, and *HTT* overlapped disease-associated traits (Fig. 1c and Supplementary Table S2). In a few cases, GWAS traits corresponded to the gene-associated disease (PD for antisense lncRNAs to *SNCA* and *LRRK2* and ALS for *C9orf72*), implying the gene and its antisense in familial and sporadic forms, respectively. In other instances, however, lncRNA-overlapping trait-associated SNPs are different from the disease caused by protein-coding mutation. *MAPT* antisense lncRNAs (in the 5′, intragenic and 3′ regions) display association to PD and, with a minor number of SNPs, to anemia and defects of the red blood cells. Trait association to diseases of the red blood cells and coagulation system is observed for antisense lncRNAs to *MAPT*, *SOD1*, and *FUS*. GWAS-associated SNPs linked to diseases of the immune system are present for antisense transcripts to *C9orf72* (inflammation and rheumatoid arthritis) and *LRRK2* (Crohn’s disease, asthma). Neurodegeneration- and immune system-unrelated traits were observed for antisense lncRNAs to *APP* (insulin resistance and type 2 diabetes), *SOD1* (esophageal cancer), and *HTT* (abnormality of the myocardium) opening interesting insights into the potential role of these lncRNAs in diseases different from those caused by mutations of the corresponding sense protein-coding gene.

To further substantiate the relevance of these antisense lncRNAs to disease traits, we used FANTOM CAT online tool and extracted sample ontology annotation enrichment. To directly compare the pattern of sample ontology to that of disease traits, we pooled the ontology terms related to the same organ and counted the number of entries for each lncRNA (Fig. 1d and Supplementary Table S3). For five of the 24 FANTOM CAT antisense lncRNAs there was a clear association between cells and tissue types and related traits (Fig. 1d and data not shown). This included brain disease traits with expression in the nervous system (antisense to *APP* and two antisense lncRNAs to *MAPT*) as well as immune-related diseases and enrichment in immune cells (antisense to *LRRK2* and *C9orf72*). The contribution of these lncRNAs to cell–trait pairs is also supported by their dynamic upregulation during iPSC (induced pluripotent stem cells) differentiation to neurons (*APP* and *MAPT* antisense) or in immune cells upon infection (*C9orf72* and *LRRK2* antisense) (data not shown). In the remaining genes, the expression enrichment did not parallel the disease traits. We observed a general enrichment in the samples of the nervous system, as somehow expected from antisense genes in *loci* responsible for familial neurodegeneration, and in cells of the immune system, but without a direct counterpart in associated traits. Considering that all selected lncRNAs are antisense to genes associated to familial forms of neurodegenerative diseases, we hypothesized that some sample ontology overlap between them may occur. Therefore, we carried out clustering analysis using the most-enriched sample ontology terms to search for commonalities, (Supplementary Fig. S1). Using tissue (UBERON) categories, antisense to *MAPT*, *APP*, and *HTT* and *LRRK2*, *C9orf72*, and *PSEN1* clustered together with nervous and immune system groups, respectively. A less stringent overlap was observed for the other lncRNA genes and using cell ontology (CL) categories.

Altogether, we showed that antisense lncRNAs to genes associated to familial forms of neurodegenerative diseases may themselves be implicated in multiple diseases, for their overlapping with trait-associated SNPs derived from GWAS studies.

It follows a gene-by-gene analysis and validation of selected examples of antisense transcripts at *loci* associated to hereditary neurodegenerative diseases. The full list of antisense TSSs described below is shown in Supplementary Table S4.

### Antisense Transcription in Genes Associated to AD

#### *APP*

**Fig. 2:**
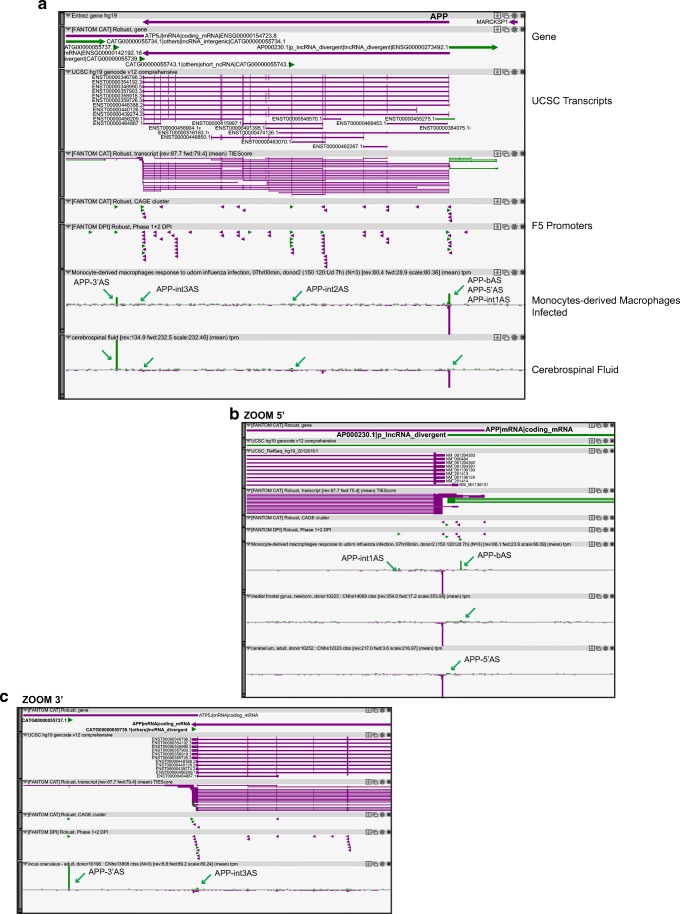
Antisense transcription in AD-associated *APP locus*. **a** Zenbu genome browser view of gene *locus* for human *APP*. Genes and transcripts are color-coded according to their orientation in the genome (+ strand, green; − strand, purple). Green arrows highlight tags of antisense transcription. Annotated UCSC transcripts are shown, with exon (thick lines) and intron (thin lines) boundaries. Antisense lncRNAs annotated in public databases are present within UCSC transcripts. FANTOM CAT functional annotation of coding and noncoding genes is shown. FANTOM5 promoters (robust DPI and robust CAT clusters) are indicated as small arrowheads, color-coded according to the direction of transcription. Arrows highlight *APP* antisense TSSs with their nomenclature. hCAGE data in infected monocyte-derived macrophages after infection (blood) and cerebrospinal fluid (brain) libraries are shown, where antisense expression is more evident. **b** Zoomed-in image of antisense transcription at the 5′ end of the *APP locus*. Monocyte-derived macrophages, medial frontal gyrus, and cerebellum libraries have been selected to better show alternative TSS usage. AP000230 antisense p-lnRNA gene is indicated. **c** Zoomed-in Zenbu genome browser view of *APP* 3′ region. FANTOM CAT annotation of antisense genes is shown. Antisense TSSs are indicated as in **a** and **b**

A complex pattern of antisense transcription is present in the locus for *APP* (Fig. 2a), with alternative TSSs at the 5′ (APP-bAS, APP-5′AS, and APP-int1AS) and 3′ (APP-3′AS) and in the intragenic region (APP-int2AS and APP-int3AS). Four noncoding genes are annotated in FANTOM CAT as promoter-associated (*AP000230.1*), short ncRNAs (*CATG00000055743.1* and *CATG00000055739.1*), and others-lncRNA (*CATG00000055737.1*). At the 5′ of *APP*, antisense transcription is evident in the region of where APP transcription starts. A series of tags are present along the main sense TSS (Fig. 2b), with some of them overlapping with all *APP* isoforms, and others overlapping only with *APP* isoforms with longer 5′ UTR, based on FANTOM CAT RNA sequencing and reciprocal S/AS TSS positions. APP-5′AS is strongly enriched in the nervous system and it is expressed in different brain regions. SNP polymorphisms in its sequence are associated to schizophrenia and type 2 diabetes. Sample ontology–disease trait pairs link APP-5′AS expression to the brain and type 2 diabetes and schizophrenia (Fig. 1, Table 3 and Fig. S1). APP-int1AS TSS displays a low level of expression, similar to that of APP-5′AS. Instead, APP-bAS, a TSS located more upstream to APP-5′AS, is selectively enriched in monocyte-derived macrophages upon infection and in various brain structures (substantia nigra, striatum, amygdala, thalamus, and cortex) mainly from newborn (Fig. 2b).

In the intragenic region, two robust promoters (APP-int2AS and APP-int3AS) represent the TSS for FANTOM CAT annotated short and long noncoding genes, respectively (Fig. 1a, c). The APP-int2AS is used in more than 60% of all FANTOM5 libraries, with the highest levels in epithelial cells upon epithelial-to-mesenchymal transition and in myoblast differentiation into myotubes and in various brain regions. A similar distribution, but at a lower level, is observed for the lncRNA APP-int3AS. Expression of both ncRNAs is dynamically regulated during iPS cell differentiation to neurons with almost 10-times downregulation after 12 days in culture. The same pattern is observed using iPS cells derived from Down Syndrome patients (data not shown).

The highest level of antisense transcription in quantitative terms is observed at the 3′ end of APP (APP-3′AS), which is present in almost all FANTOM5 libraries (> 93%). Interestingly, during iPS cell differentiation to neurons, APP-3′AS shows an opposite regulation compared to intragenic antisense ncRNAs, being upregulated at later differentiation stages (5-fold induction in Down Syndrome cells).

#### *PSEN1*

Antisense transcription is concentrated at the 5′ end of the *PSEN1* gene (Supplementary Fig. S2A) PSEN1-5′AS is a bona fide lncRNA annotated in FANTOM CAT and with epigenetic features of promoter-associated divergent transcript. PSEN1-5′AS is mainly expressed in the cells of the immune system, namely in eosinophils, neutrophils, and CD14^+^ monocytes. A low level can be measured in brain structures, such as the cerebellum and medial frontal gyrus and iPSC-derived neurons. In brain tissues, the usage of an alternative 5′ AS TSS (PSEN1-bAS) is observed, enriched in newborn structures (cortex, hippocampus, striatum). Finally, it has to be noted that an alternative, new TSS for PSEN1 is used mainly in immune cells and, to a less extent, in the brain.

#### *PSEN2*

In the *PSEN2 locus* (Supplementary Fig. S2B), a robust promoter is present in the intragenic region (PSEN2-intAS). PSEN2-intAS is expressed in a restricted number of libraries derived from adipose tissue. At the 5′ end, some antisense TSSs (permissive set) are used in the liver and brain (PSEN2-5′AS).

### Analysis of Antisense Transcription in FTD-Associated *Loci*

#### *C9orf72*

Expansion of GGGGCC exanucleotide repeat in the first intron of *C9orf72* gene has been identified as the most common pathogenic mutation in families with FTD, FTD/ALS, and ALS [38, 39]. Antisense transcription at this locus is concentrated at its 5′ end (Supplementary Fig. S3A). Three antisense TSSs (AS1, AS2, and AS3) have been recently characterized, and AS3 (or C9orf72-5′AS) has been shown to be upregulated in the brain of patients with FTD [42]. We inspected FANTOM CAT to gain further insights into the genomic features of C9orf72-5′AS. C9orf72-5′AS is confirmed in FANTOM CAT annotation and shows epigenetic features of promoter-associated antisense lncRNA. Interestingly, it is more expressed in the blood than in the brain, and it is induced in CD14^+^ monocytes upon bacterial infection (TPM ranging from 50 to 150). A similar pattern of expression is shared with C9orf72-AS1 and C9orf72-AS2, but AS2 is used in a few libraries and at a low level, while AS1 is specifically enriched in eosinophils and neutrophils (Supplementary Fig. S3A, zoom).

Using FANTOM CAT assembly, we also identified a new antisense transcript to the *C9orf72* gene, located at its 3′ end (C9orf72-3′AS). This is a poorly characterized RNA, with yet unknown coding potentials. Based on its anatomy and relative TSS positions, C9orf72-3′AS overlaps some *C9orf72* isoforms at the 3′ end and to MOB3B mRNA at the 5′ end (Supplementary Fig. S3A and data not shown). CAGE data indicate expression of C9orf72-3′AS in the cells of the immune system, mainly in infected monocytes, macrophages, and cord blood-derived lymphoblastoid cells.

#### *GRN*

No antisense transcripts are known so far within the *GRN* gene. FANTOM5 analysis fails to identify any robust promoter at this *locus*, suggesting that any antisense transcript would be expressed at a low level and used in a restricted number of libraries. Indeed, 39 antisense TSSs can be detected using the permissive set of FANTOM5 promoters. Among these, two antisense promoters at the 5′ of *GRN locus* are more expressed than others and mainly in blood libraries. At the 5′, antisense tags precede the main sense TSS (Supplementary Fig. S3B), suggesting the existence of a bidirectional promoter (GRN-5′AS). An additional TSS overlaps the first intron, thus identifying a GRN-intAS transcript. GRN-5′AS and GRN-intAS are both expressed in monocytes upon infection, while only GRN-5′AS shows some expression in the brain (Supplementary Fig. S3B).

#### *MAPT*

**Fig. 3:**
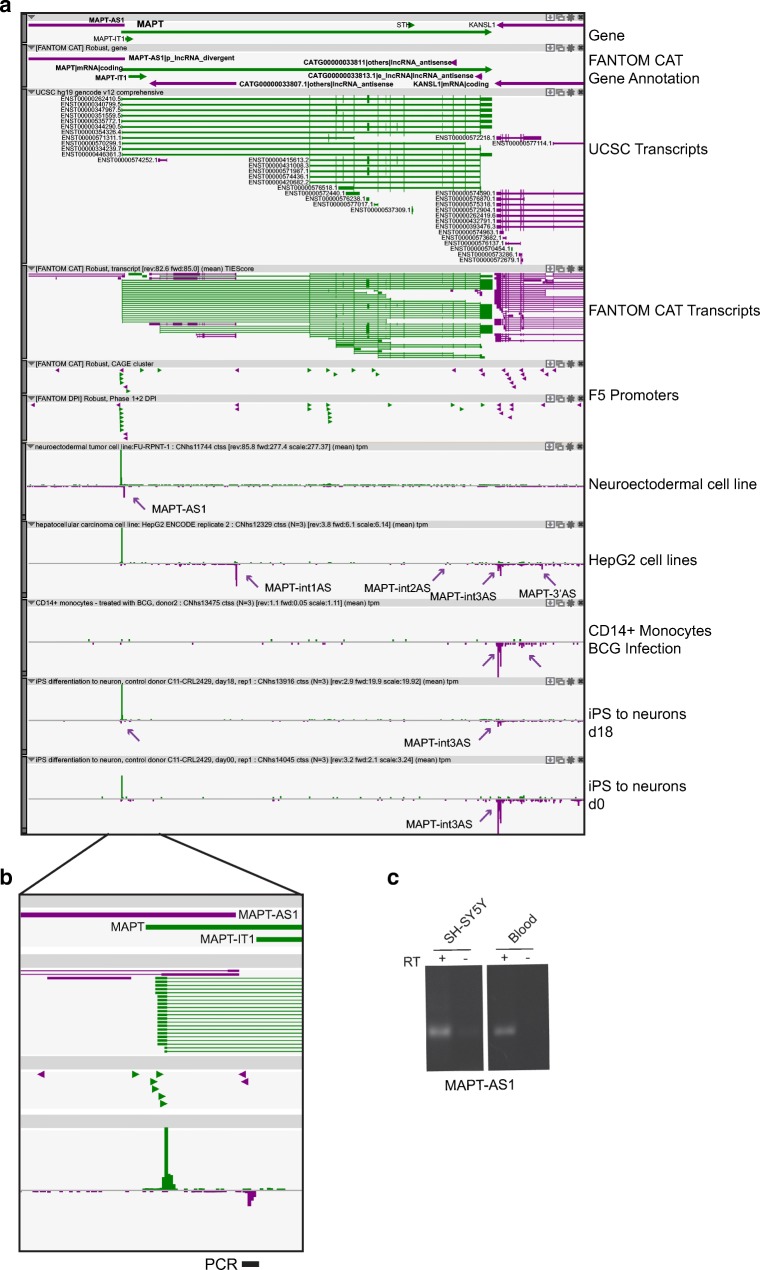
Antisense transcription in FTD-associated *MAPT*. **a** Zenbu genome browser view of gene *locus* for human *MAPT*. FANTOM CAT assembly indicates functional categories of antisense lncRNA genes. Annotated (UCSC transcripts) and newly identified (FANTOM CAT transcripts) transcripts in sense (green) and antisense (purple) orientation are shown. FANTOM5 promoters (F5 promoters) are indicated by green and purple arrowheads, according to their orientation on the genome. A series of FANTOM5 libraries was selected to highlight the complexity of antisense transcription at this *locus* and the selectivity of alternative TSS usage in different cell types. iPS cells at d0 and d18 of differentiation to neurons indicate dynamic regulation of MAPT-int3AS expression. **b** Zoomed-in image of *MAPT* locus at its 5′ end, with genomic position of primers used for validation. **c** PCR validation of MAPT-5′AS expression in human neuroblastoma cell line (SH-SY5Y) and in human blood (Blood)

A series of overlapping sense and antisense genes are annotated in the *locus* for *MAPT* (Fig. 3). Of these, four are categorized as antisense lncRNAs in FANTOM CAT: a promoter-associated lncRNA gene at the 5′ (*MAPT-AS1*), two others-lncRNA genes in the intragenic region (*CATG00000033807.1* and *CATG00000033811.1*), and an enhancer-associated lncRNA gene toward the 3′ end of the locus (*CATG00000033813.1*) (Fig. 3 and Table 3). All these *MAPT* antisense lncRNA genes have been identified in GWAS studies and represent susceptibility risk factors for PD. In addition, the same lncRNA *loci* are linked to diseases outside the brain and, in particular, to autoimmune diseases and abnormalities of red blood cells (Fig. 1c). However, the brain function of these lncRNAs seems to dominate: sample ontology analysis indicates that their expression is particularly enriched in categories of the nervous system and in neurons, rather than in immune cells or in other blood cell types (Fig. 1d and Supplementary Fig. S1). The antisense gene at the 5′ represents a promoter-associated lncRNA gene. Expression of *MAPT-AS1* is driven by three alternative TSSs giving rise to MAPT-AS1 transcripts (Fig. 3a, b). Their usage is restricted to 68 libraries out of almost 2000 in FANTOM5 collection and is highest in neuroectodermal cell lines and in brain tissues. MAPT-AS1 lncRNA (previously referred to as NR_024559) is a canonical 5′ head-to-head divergent transcript, with an intronic TSS and partially overlapping with MAPT. We validated the expression of MAPT-AS1 by RT-PCR experiments in SH-SY5Y neuronal cell line and human whole blood, using primer pairs designed around the TSS (Fig. 3c).

In the intragenic region, MAPT-int1AS (*CATG00000033807.1*) is almost exclusively expressed in the liver and in hepatocellular carcinoma cell lines HepG2, while MAPT-int2AS (*CATG00000033811.1*), albeit annotated, cannot be measured after stringent cutoff (Fig. 3a). MAPT-int3AS is also expressed at low levels in the brain. Interestingly, MAPT-int3AS is inversely co-regulated with *MAPT* mRNA during iPS cell differentiation into neurons (Fig. 3a).

No transcripts are annotated at the 3′ of *MAPT* locus. In this region, a set of robust cluster indicates the expression of a putative new antisense transcript (MAPT-3′AS). Different from the brain-specific nature of the other antisense TSSs, MAPT-3′AS is mainly expressed in epithelial cells, in embryonic cells, and in cells of the immune system (higher in CD4^+^ and CD8^+^ T lymphocytes).

### Antisense Transcripts Associated to Genes Causative of Hereditary PD

#### *SNCA*

**Fig. 4:**
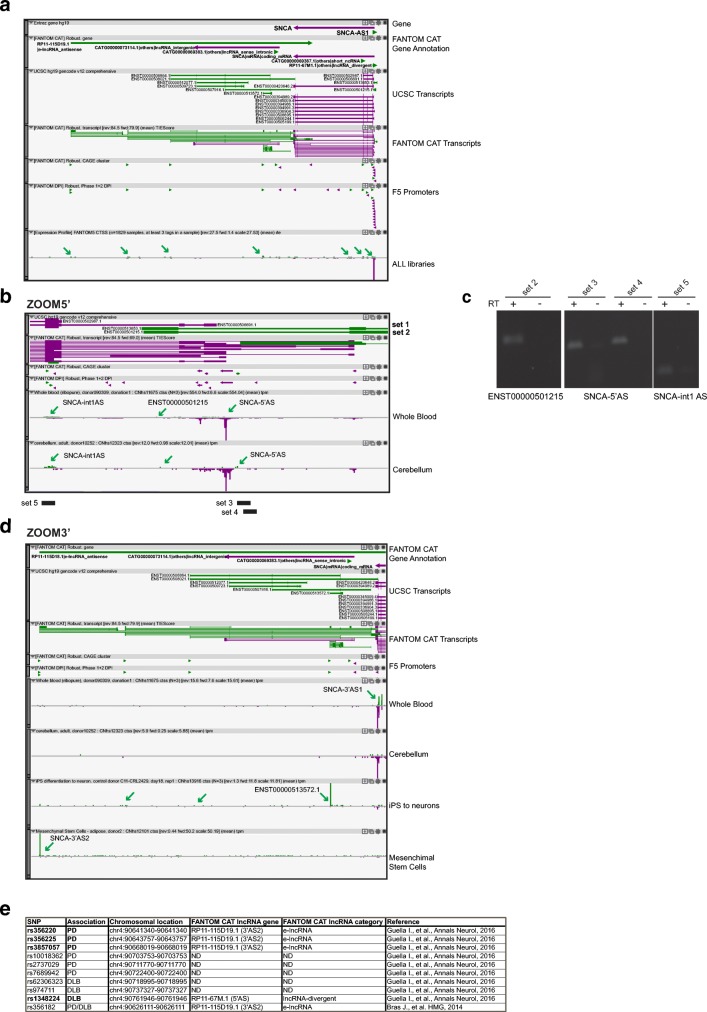
Antisense transcription at *SNCA* gene *locus*. **a** Zenbu genome browser view of PD-associated *SNCA locus*. α-Synuclein is expressed from the minus strand of the genome (purple arrow). Annotated (UCSC) and FANTOM5-derived (FANTOM CAT) assembly of genes and transcripts is shown. A series of green arrows indicates antisense transcription in pooled FANTOM5 libraries (F5 ALL). **b** Zoomed-in image of the genomic region at the 5′ end of *SNCA*. Transcripts for α-synuclein (purple) and antisense lncRNAs (green) are indicated. hCAGE peaks indicate expression of SNCA-5′AS, ENST00000501215, and SNCA-int1AS (green arrows). Sets of primers (set 1 to set 5) used for PCR validation are shown. PCR amplicons encompassing the region of interest are shown at the bottom (black bars). **c** PCR validation of α-synuclein antisense transcripts in the blood. Validated annotated (ENST00000501215) and newly discovered antisense transcripts (SNCA-5′AS and SNCA-int1AS) are indicated at the bottom. **d** Zoomed view of FANTOM5 data at the 3′ of *SNCA* locus. FANTOM CAT antisense genes are highlighted in bold. Annotated α-synuclein transcripts (purple) and antisense lncRNAs (green) are shown. Tracks with hCAGE data are selected to show usage of alternative TSSs in different samples. **e** Overlay of single nucleotide polymorphisms (SNPs) reported for the *SNCA* with FANTOM CAT annotation of antisense lncRNAs in this region. Disease association is shown (PD, Parkinson’s disease; DLB, dementia with Lewy bodies; ND, not detected). Reference to the original study is indicated

Antisense transcription is present across the whole locus of α-synuclein (*SNCA*) (Fig. 4a), at the 5′ and 3′ extremities, and in the intragenic region. At the 5′ end, a lncRNA gene is annotated in public databases (*SNCA-AS1*) and confirmed by FANTOM CAT (others-lncRNA_antisense). We found three additional noncoding genes antisense to *SNCA*: a short ncRNA in the intragenic region and two lncRNA genes at the 3′, giving rise to several alternative isoforms partially overlapping with α-synuclein mRNA. All these antisense noncoding genes are contained in the genomic region that is duplicated [61] and triplicated [62] in α-synuclein genetic cases of PD. At the 5′ end, two alternative transcript variants exist (ENST00000513653 and ENST00000501215) of a lncRNA gene validated in FANTOM CAT. ENST00000513653 and ENST00000501215 are oriented to form a S/AS pair 5′ head-to-head divergent from α-synuclein. Based on reciprocal TSS position and RNA sequencing data, antisense transcripts overlap with α-synuclein variants with longer 5′ UTR. When we inspected FANTOM5 libraries for expression of these transcripts, we detected only few tags corresponding to the annotated site of transcription initiation, classified in the permissive promoter set (Fig. 4b and data not shown). To validate the expression of annotated AS transcripts, we designed intron-spanning primers to amplify ENST00000513653 (set 1) and ENST00000501215 (set 2). Using set 2, we experimentally validated the expression of AS lncRNA ENST00000501215 in human whole blood (Fig. 4c).

In the 5′ region, we identified additional TSSs that do not map with annotated transcripts, but find validation in CAGE, RNAseq, and FANTOM CAT assembly. SNCA-5′AS TSS drives the expression of transcripts overlapping only with longer isoforms of sense α-synuclein mRNA. Tags for SNCA-5′AS can be measured in several brain areas including the cerebellum, amygdala, medulla oblongata, and cortex and in the blood. Using PCR primers designed around AS TSS sequences and positioned in nonexonic portion of sense mRNA (sets 3 and 4), we validated the expression of SNCA-5′AS in human whole blood (Fig. 4c). An additional AS TSS is located around the exon II of α-synuclein at the intron–exon boundary (SNCA-int1AS) and overlaps with all isoforms of α-synuclein (Fig. 4b). FANTOM CAT assembly confirms that SNCA-int1AS is a novel antisense lncRNA to α-synuclein. Expression of SNCA-int1AS in the blood is highest in whole blood samples, with levels ranging from 24 to 90 TPM. Indeed, blood and reticulocyte ontology terms are enriched for this antisense lncRNA. Moreover, specific expression tags are found in dendritic cells and monocyte-derived macrophages in response to LPS treatment (11.65 TPM) and infection by udorn influenza virus (5.27 TPM). In the brain, several regions show some level of SNCA-int1AS expression, including the substantia nigra and the cerebellum (Fig. 4b). Using whole blood human sample and PCR primers designed around its TSS, we experimentally validated the expression of this previously unidentified AS transcript (Fig. 4c).

Finally, we identified robust promoters for a series of annotated processed transcripts with noncoding features at the 3′ end of α-synuclein gene (Fig. 4d). The first of them, SNCA-3′AS1, belongs to the permissive set of promoters and its expression is enriched in samples from the whole blood. An annotated transcript, ENST00000513572, is positioned 3′ to SNCA-3′AS1 and overlaps with α-synuclein isoforms with longer 3′ UTR. The highest tag counts for ENST00000513572 were found in hematopoietic stem cells, cord-blood derived cells, and iPSC-derived neurons. Finally, an e-lncRNA (SNCA-3′AS2) is expressed at the 3′ end of the *locus*. This antisense lncRNA is enriched in endothelial and mesothelial cells, implicating a potential role in these cells.

Since *SNCA* is considered a susceptibility factor also for sporadic PD, we monitored newly identified antisense lncRNA genes for their association to diseases. Intersection with publicly available GWAS studies indicated that lncRNAs at both 5′ and 3′ extremities represent risk factors for PD (all antisense lncRNAs) and prion disease (3′ antisense only). Since *SNCA* variants have been recently attributed to PD-related dementias, we overlaid SNPs associated to these diseases to FANTOM CAT lncRNA annotation. We found that PD-associated SNPs are enriched in the 3′ e-lncRNA, while Dementia with Lewy Body phenotype maps to the 5′ antisense lncRNA (Fig. 4e).

#### *PRKN*

*PRKN*, with *MAPT*, is the neurodegenerative disease-associated gene with the most abundant number of antisense genes and alternative antisense TSSs (Supplementary Fig. S4A). First, the protein-coding *PRKN* co-regulated gene (*PACRG*) is located in antisense orientation to *PRKN* and is organized in a divergent 5′ head-to-head configuration (Supplementary Fig. S4A). Expression of *PRKN* and *PACRG* is strongly co-regulated, with relatively high levels in the brain and almost undetectable levels in the blood. Five additional antisense genes are annotated in FANTOM CAT, and four of them are classified as lncRNAs (Table 3). These antisense lncRNA genes are located in the intragenic region of *PRKN*, with two of them displaying epigenetic features of enhancer-associated lncRNAs. While expression is enriched in ontology terms related to brain functions (Fig. 1 and Supplementary Fig. S1 and Supplementary Table S2), none of them has been found associated to brain diseases. Some linkage is observed to metabolic disorders, artherosclerosis, and impairment of the auditory system (Fig. 1). TSSs of these lncRNAs suggest very specific expression and alternative usage. PRKN-int1AS e-lncRNA is enriched in iPSC-derived neurons. PRKN-int2AS TSS is almost exclusively expressed in the pineal gland at quite high levels (30 TPM), with lower expression also in the retina and the eye (5–9 TPM). A similar pattern of restricted expression is also observed for the other antisense TSSs: PRKN-int3AS and PRKN-int4AS in cancer cell lines, PARKN-int5AS in ARPE-19 EMT cells induced with TGFβ and TNFα, and PARKN-3′AS1 in CD14^+^ monocytes.

#### *PINK1*

Inspection of the *PINK1 locus* (Supplementary Fig. S4B) reveals the presence of an antisense lncRNA transcript, previously annotated in public databases (ENST00000451424). This has been recently described as *PINK1* natural antisense transcript (naPINK1 or PINK1-AS) [37]. FANTOM CAGE and sequencing data confirm the anatomy of this antisense lncRNA. PINK1-AS contains three repetitive elements of the Alu family that are positioned at the 5′ (position 70), middle (position 2441), and 3′ end (position 4143) of the transcript and outside a putative small ORF of 112 amino acids (positions 1496–1834). Analysis of PINK1-AS expression indicates regulation during iPS cell differentiation into neurons (from 2.9 to 0.5 TPM). In the blood, PINK1-AS is expressed at low level in conventional memory CD4^+^ T cells.

A clear peak of antisense transcription is also present in a nonannotated region at the 5′ end of the *PINK1* gene (Supplementary Fig. S4B). PINK1 5′ antisense TSS is located within the first intron of the sense mRNA, identifying a novel 5′ head-to-head divergent natural antisense transcript (PINK1-5′AS). Expression of this novel antisense RNA to PINK1 is generally low and restricted to 23 libraries in the FANTOM5 dataset, mostly in cells of the immune system, neutrophils (2.7–3.5 TPM), and CD8^+^ and CD4^+^ T cells (naïve, memory, and regulatory).

#### *PARK7*

**Fig. 5:**
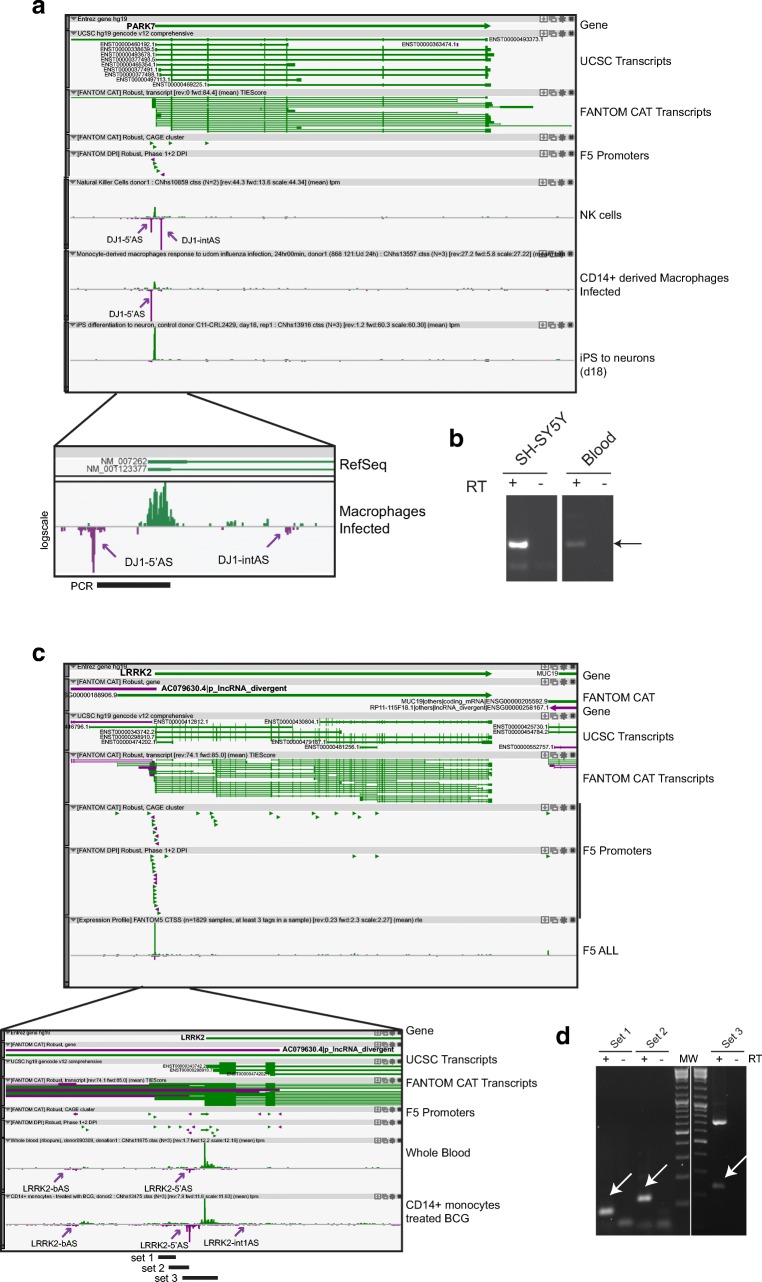
Antisense transcription in *LRRK2* and *PARK7 loci* associated to hereditary PD. **a** Zenbu genome browser view of hCAGE data in brain and blood libraries in *PARK7 locus*. No annotated transcripts and no FANTOM CAT antisense gene assembly are present at this gene. FANTOM5 promoters (F5 promoters) are indicated by green and purple arrowheads, according to their orientation on the genome. Purple arrows show tags for DJ1-5′AS and DJ1-intAS transcripts. Zoomed image show the magnification of AS TSSs and the genomic position of primers used for PCR validation (expected PCR fragment is indicated, black bars). **b** Validation of DJ1-5′AS expression in human neuroblastoma cells (SH-SY5Y) and in human whole blood (blood) by RT-PCR. Arrow indicates DJ1-5′AS-specific band (verified upon cloning and sequencing). **c** Antisense transcription in *LLRK2 locus*. *LRRK2* protein-coding and antisense p-lncRNA (*AC079630.4*) genes are highlighted in bold. Annotated (UCSC) and FANTOM5 (FANTOM CAT) transcripts are shown. Zenbu track shows sense/antisense transcription in pooled FANTOM5 libraries (F5 ALL). Indent shows zoom-in of the 5′ region. Purple arrows highlight TSSs for LRRK-bAS, LRRK2-5′AS, and LRRK2-int1AS in selected brain and blood libraries. Sets of PCR products are shown in black. **d** Validation of LRRK2-5′AS expression in whole blood by RT-PCR. Primers used for validation and amplicons are indicated. White arrows indicate PCR bands corresponding to LRRK2-5′AS, as verified by cloning and sequencing

The *PARK7 locus*, encoding for DJ-1 protein, does not present any annotated antisense transcripts (Fig. 5a). However, we found evidence for robust antisense promoters at the 5′ end of DJ-1 main TSS. Usage of these AS promoters is more evident in blood-derived libraries than in brain samples. Two independent TSSs can be identified (Fig. 5a, zoom). The first TSS is positioned 5′ to sense TSS (DJ1-5′AS), giving rise to a 5′ bidirectional antisense transcript. The second is located in the first intron of DJ-1 mRNA and identifies a new 5′ head-to-head divergent natural antisense transcript (DJ-1 intAS). In the brain, DJ1-5′AS is expressed at low level in the caudate nucleus, cerebellum, and hippocampus (TPM values 1.9–2.1) and enriched in iPS cells differentiating into neurons. DJ1-int1AS is virtually absent from brain tissues and brain-derived primary cells. On the contrary, DJ1-5′AS and DJ1-intAS are both highly expressed in the immune system, with TPM values as high as 40, and differentially regulated in various cell compartments and during immune activation. DJ1-5′AS is highly induced (more than 20-fold) in monocyte-derived macrophages responding to viral and bacterial infection (Fig. 5a). Similarly, selective stimulation of mast cells causes a 10-time fold increase in DJ1-5′AS levels. A milder effect is observed in CD14^+^ monocytes after infection (1.5–2.0-fold induction). DJ1-5′AS is also expressed at high levels in NK cells (20 TPM), CD8^+^ and CD4^+^ T cells (in the range of 15 and 8 TPM, respectively), basophils, mast cells, and dendritic cells. Much lower levels are measured in B cells (1.5–2.0 TPM). DJ1-intAS is strongly expressed in nonactivated cells in the immune system, such as NK cells (51 TPM), in CD8^+^ (25 TPM) and CD4^+^ (10 TPM) T lymphocytes, in B cells (12–16 TPM), and in uninfected CD14^+^ monocytes (10 TPM).

RT-PCR validation of antisense transcription in the 5′ region of DJ-1 was successfully carried out from human neuroblastoma SH-Y5Y cells and whole blood (Fig. 5b).

#### *LRRK2*

Antisense transcription is concentrated at the 5′ end of *LRRK2* (Fig. 5c). Three alternative TSSs in this region (LRRK2-bAS, LRRK2-5′AS, and LRRK2-int1AS) provide transcription initiation for a promoter-associated antisense lncRNA gene. This FANTOM CAT annotated gene is highly enriched in hematopoietic cells of the immune system and associated to a number of autoimmune diseases (Crohn’s disease, multiple sclerosis, glomerulosclerosis) and immune-related dysfunctions (asthma) (Fig. 1, Table 3, and Supplementary Fig. S1 and Table S2).

LRRK2-bAS is an antisense lncRNA annotated in public databases (ENST00000412812). It does not overlap with any annotated LRRK2 mRNA variants, thus representing as bidirectional transcript. This 585 base pair-long lncRNA presents four processed exons, one SINE (17–152) and two LTR (153–288; 286–412) repetitive elements. It is expressed in eosinophils (TPM 4.2) and, to less extent, in neutrophils and CD14^+^ monocytes upon infection (1.5–2.0 TPM). Similar to LRRK2-bAS, LRRK2-5′AS is strongly enriched in cells of the immune system. Neutrophils show the highest levels of LRRK2-5′AS expression as compared to both blood- and brain-derived cells and tissues (24 TPM). In the blood, LRRK2-5′AS expression is also detected in specific subsets of purified immune cells (monocytes, B cells, eosinophils, mast cells, and CD8^+^ T cells), while virtually absent in CD4^+^ T cells and CD34^+^ bone marrow precursors. Treatments with γ-interferon, LPS, influenza virus, and bacillus tuberculosis increase LRRK2-5′AS levels in CD14^+^ monocytes. In brain tissues, LRRK2-5′AS is more expressed in globus pallidus (1.9 TPM) and in striatal areas (putamen and caudate nucleus, 0.7–1.1 TPM). Antisense transcription in the 5′ region of LRRK2 has been validated in human whole blood by RT-PCR with primers mapping around hCAGE tags (Fig. 4c, d).

#### *VPS35*

Bidirectional antisense transcription is evident at the *VPS35 locus*, where the *ORC6* protein-coding gene is annotated (Supplementary Fig. S5). A short noncoding RNA is also identified in FANTOM CAT (VPS35-AS), but its expression is limited to few libraries, such as CD14^+^ monocytes.

### Antisense Transcription in ALS

Four genes linked to familial cases of amyotrophic lateral sclerosis were investigated for antisense transcription: *SOD1*, *FUS*, *TARDBP*, and *UBQLN2* (Supplementary Fig. S6). All four genes are positioned in genomic location almost completely devoid of any other annotated gene.

#### SOD1

SOD1 is highly expressed in the brain and blood. Peaks of antisense transcription can be detected at a lower level, as expected. An antisense lncRNA ENST00000449339 is annotated at the 5′ of SOD1 (Supplementary Fig. S6A), and FANTOM5 data confirm its promoter (F5 promoters, permissive DPI set). Its TSS is located upstream of SOD1 transcription initiation, thus resulting from the activity of a bidirectional promoter. ENST00000449339 is expressed mainly in brain libraries, such as the thalamus, hippocampus, striatum, and cortex (TPM values 2.1–0.5). In addition to the annotated lncRNA, we identify promoters for an additional antisense transcript in the first intron of SOD1 (SOD1-5′AS). SOD1-5′AS represents a previously unidentified promoter-associated lncRNA. SOD1-5′AS is mainly expressed in the brain (Supplementary Fig. S6A), with higher levels in the globus pallidus, locus coeruleus, corpus callosum, medulla oblongata, spinal cord, and substantia nigra (TPM values ranging around 3.5). In the blood, SOD1-5′AS expression is enriched in dendritic cells, mast cells, and monocyte-derived macrophages upon LPS treatment and infection with influenza virus. Interestingly, genetic variations in SOD1-5′AS gene have been linked to cancer and sickle cell anemia (Fig. 1). Tags of antisense transcription can also be observed in SOD1 intragenic region (SOD1-int1AS and SOD1-int2AS) in both brain and blood libraries (Supplementary Fig. S6A). SOD1-int2AS is annotated in FANTOM CAT as e-lncRNA, and it is expressed in iPS cells undergoing neuronal differentiation.

#### FUS

FUS is a ubiquitously and highly expressed gene and virtually all hCAGE libraries in FANTOM5 collection display some degree of FUS mRNA expression. AS transcription at this *locus* is detected at a much lower level and in few libraries (Supplementary Table S1). Three antisense lncRNAs to *FUS* have been identified by CAGE analysis, RNA sequencing, and accurate 5′ end annotation (Supplementary Fig. S6B). FUS-5′AS is positioned antisense to the first intron, generating a 5′ head-to-head AS transcript overlapping with the AUG region of *FUS* mRNA. FUS-5′AS is expressed mainly in CD8^+^ T cells in the blood and in iPSC-derived neurons. Genomic and epigenetic features indicate that FUS-5′AS is novel antisense lncRNA associated to the promoter of *FUS* gene. FUS-int1AS and FUS-int2AS TSSs are associated to enhancer lncRNAs in FANTOM CAT and generate transcripts partially overlapping to exons 3–5 of *FUS* mRNA. Interestingly, both lncRNAs are identified in a number of GWAS studies for autoimmune diseases, including systemic sclerosis, Grave’s disease, and lupus erythematosus (Fig. 1).

#### TARDBP

No antisense transcripts are annotated in this *locus*. Using FANTOM5 data, we identify an antisense TSS centered on the main sense promoter, suggesting a 5′ head-to-head divergent natural antisense transcript (TARDBP-AS) (Supplementary Fig. S6C). The genomic and epigenetic features indicate that TARBP-AS belongs to the category of promoter-associated divergent lncRNAs. TARDBP-AS is expressed at a low level (1.5–0.5 TPM) in various human cell lines and in primary cells of the immune system (CD4^+^ and CD8^+^ T cells, CD14^+^ monocytes).

#### UBQLN2

Antisense promoters (UBQLN2-int1AS and UBQLN2-int2AS) are present in the intragenic region of *UBQLN2* and used in the brain and blood (Supplementary Fig. S6D). UBQLN2-int1AS is annotated in FANTOM CAT as promoter-associated antisense lncRNA. Its expression is enriched in pineal body and in NK cell ontology terms, suggesting a possible function in the brain and blood (Supplementary Table S2 and Supplementary Fig. 1). UBQLN2-int2AS is a small ncRNA whose expression is detected only in few libraries in the brain (occipital cortex, pons, and insula).

### Antisense Transcription in HD

#### *HTT*

HD is caused by dominant expansion of CAG repeats within the *HTT* gene. A lncRNA antisense to *HTT* (HTT-AS) has been recently identified and implicated in the regulation of *HTT* gene expression [35]. It forms a 5′ head-to-head divergent pair, overlapping with the CAG expansion region and the 5′ UTR of HTT mRNA (Supplementary Fig. S7). We confirmed the expression of HTT-AS RNA in the brain, in a wide number of brain areas and in brain-derived primary cells. Interestingly, the highest level of expression is measured in neuronal stem cells (2.4–2.9 TPM), implicating HTT-AS in neuronal differentiation. Furthermore, we could demonstrate expression of HTT-AS in the blood, especially in monocyte-derived macrophages upon infection (17.13 TPM) and in CD14^+^ monocytes after infection with bacillus tuberculosis, BCG (6.54–7.44 TPM).

Other tags of antisense transcription can be observed throughout the extension of *HTT* intragenic region (permissive promoter set). Further analysis will be required to validate them.

### Quantitative Expression of Selected Natural Antisense Transcripts and Corresponding Sense Genes in Human Tissues

**Fig. 6:**
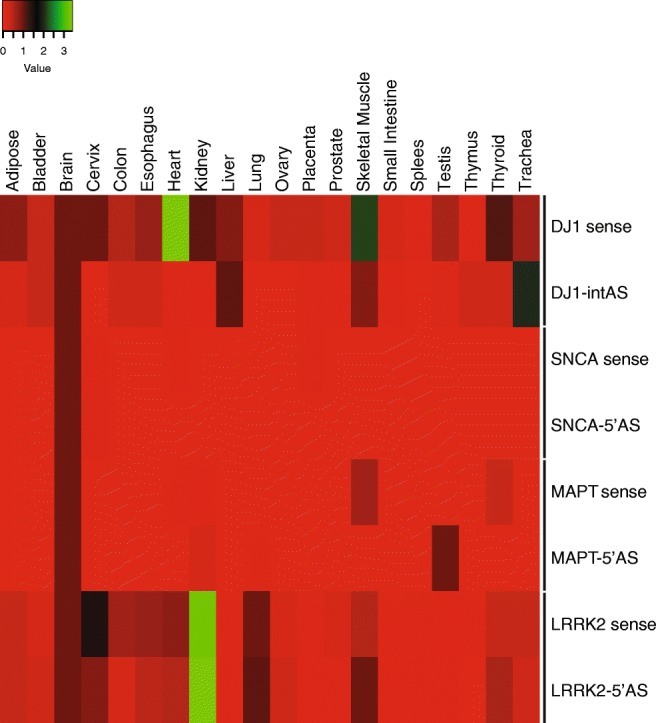
Quantitative expression of S/AS pairs for *LRRK2*, *SNCA*, *MAPT*, and *PARK7/DJ-1* in human tissues. qRT-PCR was performed in duplicate on a panel of 20 human tissues derived from three independent subjects. Tissue types are indicated on top, genes on the right. Heat map graphical representation of rescaled normalized fold expression using the ΔΔCt method. For each gene, expression in the brain is used as a reference and set to 1. Value bar shows color coding (red–green) of normalized expression.

To characterize the expression of these novel natural antisense transcripts across a wider panel of human tissues, we took advantage of commercially available RNA from human organs and carried out quantitative RT-PCR. A subset of natural antisense transcripts was chosen among those whose expression we previously validated experimentally (MAPT-5′AS, SNCA-5′AS, DJ1-intAS, and LRRK2-5′AS). Expression of the overlapping sense genes was also analyzed. As a cross-reference value of expression, we considered as 1 the transcript levels measured in the brain. First, we confirmed FANTOM5 data of antisense transcription to neurodegeneration-associated genes in the brain (Fig. 6). Each antisense shows a specific tissue distribution. Interestingly, expression of AS transcripts is generally higher in the brain than in other tissues, with the exception of LRRK2-5′AS in the kidney and DJ1-intAS in the trachea. All antisense RNAs are expressed in more than one tissue, with MAPT-5′AS and SNCA-5′AS being the most restricted transcripts. LRRK2-5′AS is highly expressed in the kidney and similarly expressed in the brain, cervix, lung, and skeletal muscle. DJ1-intAS expression in the liver and skeletal muscle is comparable to that in the brain. Expression of sense mRNA is co-regulated in all tissues with the corresponding antisense transcript in *SNCA* and *LRRK2* genes. In contrast, some degree of discordant expression is observed for *MAPT* in skeletal muscle, thyroid, and testis. The DJ-1S/AS pair shows more evident discordant expression, with increased expression of sense mRNA in 75% of the tissues relative to the brain and enriched AS in the thymus and trachea (Fig. 6).

## Discussion

Natural antisense transcripts represent a group of poorly characterized RNAs that originate from the opposite DNA strand to another gene. The most common form of antisense transcription is where an antisense lncRNA forms a S/AS pair with a protein-coding mRNA [4, 6]. As a consequence, the study of antisense transcription is unequivocally linked to the functional annotation of lncRNAs. Despite the enormous advances through large-scale genomic projects, the field is still far from completion. Here we provide an atlas of antisense transcription at *loci* associated to familial forms of neurodegenerative diseases. We are fully aware that our analysis is far from being complete. Several additional genes are known to be causative of familial forms of the neurodegenerative diseases discussed here as well as of others not included in our analysis (such as different forms of familial ataxias, just to cite a few). However, we believe that our collection covers the most relevant genes involved in pathogenic mechanisms (beyond familial forms) and the largest number of patients suffering from the hereditary forms of these diseases. Notwithstanding these limitations, the survey of almost 2000 human hCAGE libraries from major human primary cell types and tissues for 17 genes represents the first systemic-like approach to dissect antisense transcription within the brain and beyond. The expression profiles derived by accurate 5′ mapping, using CAGE, combined with lncRNAs annotation, using FANTOM CAT, pictures a complex architecture of the analyzed *loci*. We describe over 400 promoters associated to antisense transcripts. Only a limited number of the identified transcripts are annotated in public databases, while an additional fraction finds independent evidence in FANTOM CAT, often with the identification of alternative TSSs for transcript initiation. A total of 24 antisense lncRNAs are identified.

FANTOM CAT annotation indicates enrichment for divergent promoter-associated transcripts (p-lncRNA-divergent). This is in line with the intrinsic bidirectionality of transcription initiation, as recently established [63, 64] and now supported by accurate annotation of 5′ ends [6]. Our analysis suggests that, at least in genes associated to neurodegenerative diseases, bidirectional promoters are tightly regulated in different cell types and upon external stimuli. In *LRRK2*, *DJ-1*, and *PSEN1*, for example, promoter-associated antisense lncRNAs are more expressed in blood libraries than in the brain, and within blood cells, their expression is strongly activated upon infection. Intriguingly, *LRRK2*-5′*AS* has a strong linkage to autoimmune diseases, in particular Crohn’s disease and multiple sclerosis, and its expression is associated to neutrophils.

In recent years, the concept of neuroinflammation has gradually expanded to include neurodegenerative diseases as neuroinflammatory conditions. The involvement of inflammatory cells [65–68], receptors [69–71], and cytokines [72] has been demonstrated for AD, ALS, and FTD.

The discovery that antisense lncRNAs to *loci* associated to hereditary forms of the major neurodegenerative diseases are specifically expressed in cells of the innate immune system may provide cues on how mutated alleles may contribute to the pathology in non-neuronal cells.

The relevance of antisense lncRNAs described here within and outside the brain is further supported by the intersection with GWAS data. We show that, at least in some selected cases, antisense lncRNAs to neurodegeneration-associated genes overlap trait-associated SNPs different from the hereditary disease caused by the sense protein-coding gene mutation, thus implicating these lncRNAs in multiple diseases. Our results are in line with recent gene-specific [73] and omics studies [74, 75], demonstrating disease association of genetic variants falling within lncRNA *loci*.

The identification of novel antisense transcripts to familial forms of neurodegenerative diseases may also have important implications for the larger number of patients suffering from sporadic disease and disease variants. This is the case of *SNCA*. Besides *SNCA* being been implicated in families with early-onset PD, it remains the only gene strongly associated with disease susceptibility, progression, and pathology in worldwide populations. Recently, genetic variability in *SNCA* has been also attributed to the etiology of PD-related dementias, such as PD with dementia (PDD) and Dementia with Lewy bodies (DLB) [76, 77]. Specific *SNCA* variants can distinguish between symptoms of Parkinsonism and/or dementia. We found that the expressions of 5′ and 3′ *SNCA* antisense lncRNAs are strong candidates for the molecular mechanism underlying the susceptibility to DLB and PD without cognitive impairment, respectively. Moreover, considering their distinct classification (promoter-associated lncRNA and enhancer lncRNA), it is conceivable to hypothesize different modes of action, with consequences on the future opportunities for intervention.

RNA-sequencing profiling of peripheral blood leukocytes from patients with neurodegenerative diseases represents a powerful tool to discover potential peripheral biomarkers for early diagnosis. The presence of lncRNAs in biological fluids, their dysregulated expression during pathogenic processes, and their relevance in disease mechanisms, as demonstrated for *BDNF-AS* [78] and *BACE1-AS* [33], make them ideal candidates for the development of diagnostic assays. The potentials of lncRNAs for diagnostics are more advanced for cancer, where the lncRNA *MALAT1* has been included in a commercially available diagnostic kit for prostate cancer [79]. Several data have been accumulated in the recent years for AD, PD, and other neurodegenerative diseases [80–84], suggesting that lncRNAs and antisense lncRNAs have the potentials to be included in diagnostic assays for more accurate and potentially early diagnosis.

Our atlas of antisense transcription at genes associated to familial forms of neurodegenerative diseases may provide new tools to design innovative therapeutic strategies. The concurrent renaissance of RNA therapeutics for fine-tuning gene expression with inhibitory (siRNAs and ASOs) or activatory (saRNA, ASO, SINEUPs) RNA molecules has fueled the potentials of antisense lncRNAs in therapy [22]. On one hand, antisense lncRNAs themselves can be used as new therapeutic agents. This is the case of SINEUPs, a class of natural and synthetic antisense lncRNAs that promote translation of partially overlapping sense protein-coding mRNAs, thus acting as gene-specific enhancers of translation [22, 30, 85–87]. In the brain, a synthetic SINEUP targeting glial cell-derived neurotrophic factor (*GDNF*) mRNA was able to increase endogenous protein levels and ameliorate motor deficits and neurodegeneration in a mouse model of PD (Espinoza S., et al., submitted). On the other hand, antisense lncRNAs can be considered therapeutic targets. AntagoNATs, ASOs targeting endogenously expressed regulatory antisense lncRNAs, represent one of the first examples of potentially curative antisense-targeting drugs in the brain [88]. AntagoNATs were used to inhibit brain-derived neurotrophic factor antisense lncRNA (*BDNF-AS*) to increase *BDNF* expression and promote neuronal outgrowth and cell proliferation in the hippocampus [78]. A similar approach has been shown to function for *GDNF-AS* transcript, thus demonstrating the scalability of lncRNA-mediated approaches.

Recently, ASOs have been used to block the activity of an inhibitory antisense lncRNA (*SMN2-AS*) in cellular and animal models of spinal muscular atrophy (SMA). Degradation of *SMN2-AS* [89] or inhibition of its interaction with Polycomb Repressor Complex 2 (PRC2) [90] is sufficient to restore *SMN2* mRNA quantities and rescue SMA symptoms.

## Conclusions

Altogether, our results highlight the enormous complexity of gene regulation by antisense lncRNAs at any given locus and imply large potentials for diagnostic and therapeutic intervention in neurodegenerative diseases.

## Electronic Supplementary Material

ESM 1 (PDF 2542 kb)

ESM 2 (PDF 329 kb)

## Abbreviations

CAGE: Cap analysis of gene expression
S: Sense
AS: Antisense
lncRNA: Long noncoding RNA
TSS: Transcription start site
TPM: Tag per million
AD: Alzheimer’s disease
ALS: Amyotrophyc lateral sclerosis
FTD: Frontotemporal dementia
HD: Huntington’s disease
PD: Parkinson’s disease
APP: Amyloid precursor protein
PSEN: Presinilin
GRN: Granulin
MAPT: Microtubule-associated protein tau
LRRK2: Leucin-rich repeat kinase 2
PINK1: PTEN-induced putative kinase 1
PACRG: Parkin co-regulated gene
SNCA: α-Synuclein
FUS: Fused in sarcoma
SOD-1: Superoxide dismutase 1
TARDBP (TDP-43): TAR DNA-binding protein
UBQLN2: Ubiquitin-like protein ubiquilin 2
HTT: Huntingtin
