# Supplementary material for: Antisense Transcription in Loci Associated to Hereditary Neurodegenerative Diseases
Source: Mol Neurobiol. 2019 Jan 4;56(8):5392–415. doi: 10.1007/s12035-018-1465-2 (PMC6614138; doi:10.1007/s12035-018-1465-2)
Supplement: Supplementary file 1 — (PDF 2542 kb) [file 12035_2018_1465_MOESM1_ESM.pdf]

Antisense transcription in *loci* associated to hereditary neurodegenerative diseases

Silvia Zucchelli[1,2], Stefania Fedele[1], Paolo Vatta[1,3], Raffaella Calligaris[1,4], Peter Heutink[5,6,7,8], Patrizia Rizzu[5,9], Masayoshi Itoh[7,8,10], Francesca Persichetti[2], Claudio Santoro[2], Hideya Kawaji[7,8,10,11], Timo Lassmann[7,8,12,13], Yoshihide Hayashizaki[8,10], Piero Carninci[7,8,14], Alistair R.R. Forrest[7,8,15], the FANTOM Consortium and Stefano Gustincich[1,3]

A

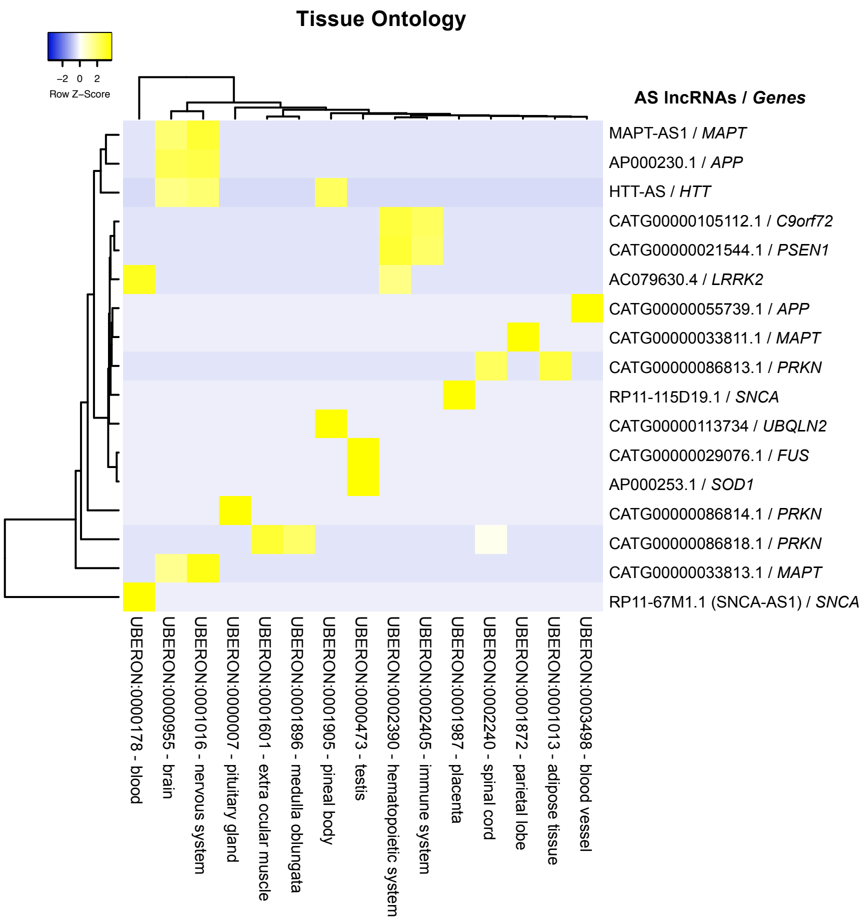

B

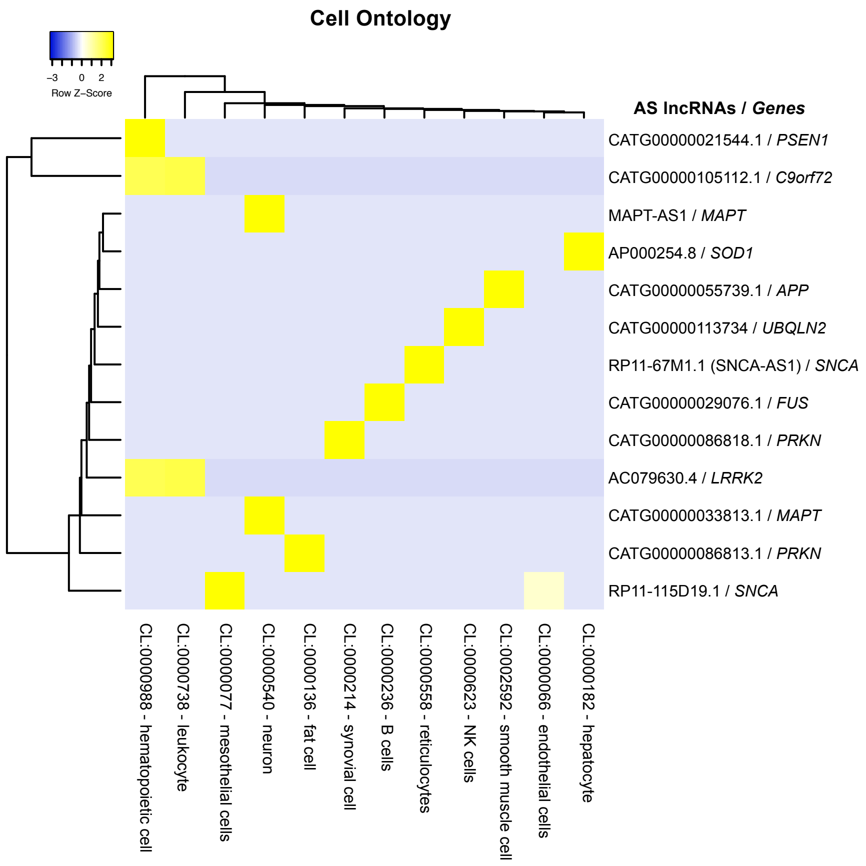

Supplementary Figure S1. Tissue and cell ontology enrichment in the expression of antisense lncRNAs to genes associated to familial forms of neurodegenerative diseases. Heat map graphical representation of fold-change expression values in specific tissue **(A)** and cell **(B)** ontology terms. AS lncRNA and protein coding gene names are indicated.

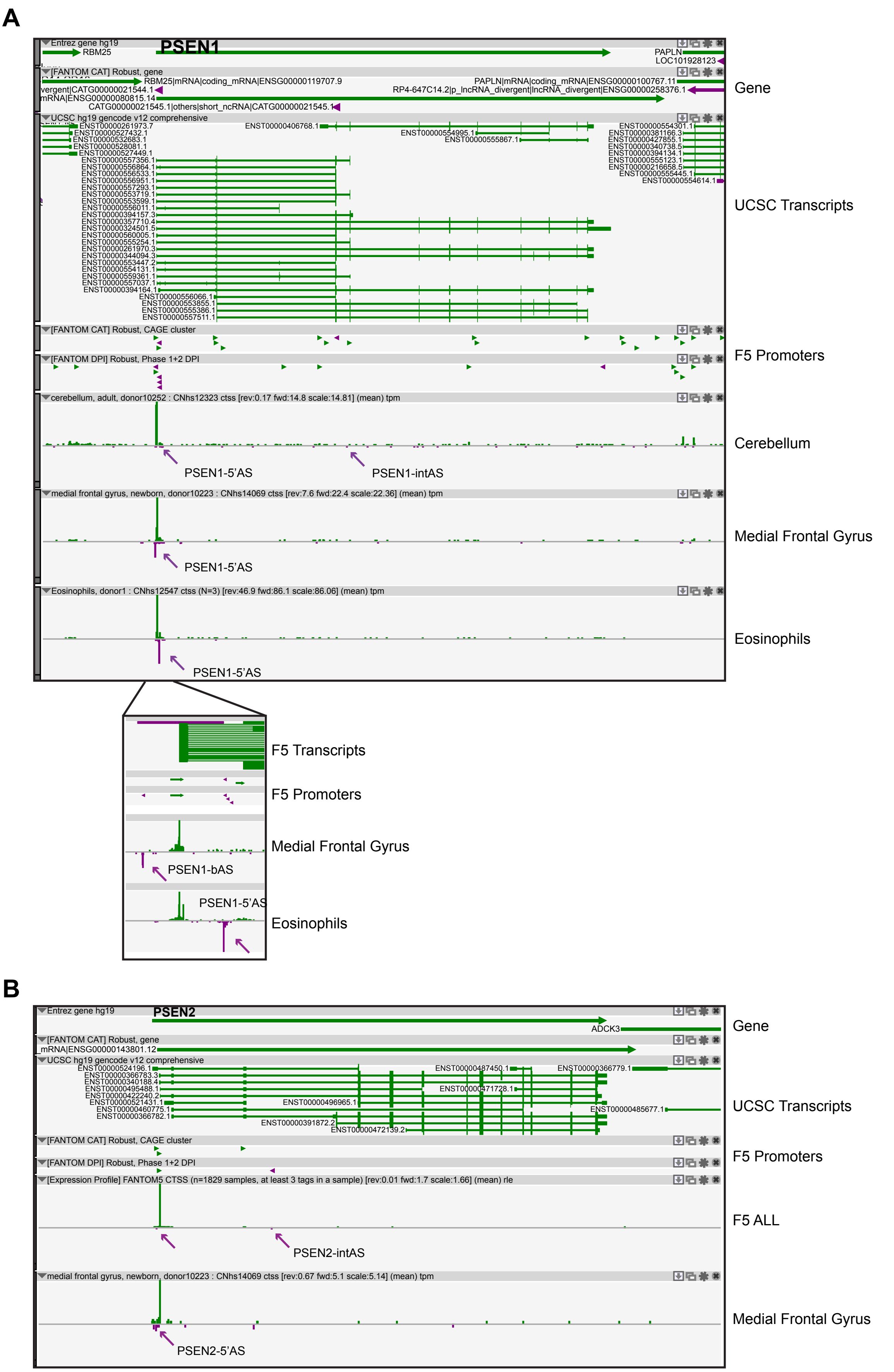

Supplementary Figure S2. Antisense transcription in genes associated to familial forms of AD. **A)** Zenbu Genome Browser view of hCAGE data in *PSEN1 locus*. FANTOM5 promoters (F5 promoters) are indicated by green and purple arrow-heads, according to their orientation on the genome. Antisense transcription in cerebellum, medial fronta gyrus and eosinophils libraries are shown. Purple arrows highlight PSEN1-5'AS and PSEN1-intAS. Zoomed image shows TSSs at the 5' region (PSEN1-bAS and PSEN1-5'AS) **B)** Zenbu Genome Browser view of *PSEN2 locus*. hCAGE data in all FANTOM5 data (F5 ALL) and in the medial frontal gyrus newborn library are shown. Purple arrows indicate AS TSSs (PSEN-5'AS and PSEN-intAS).

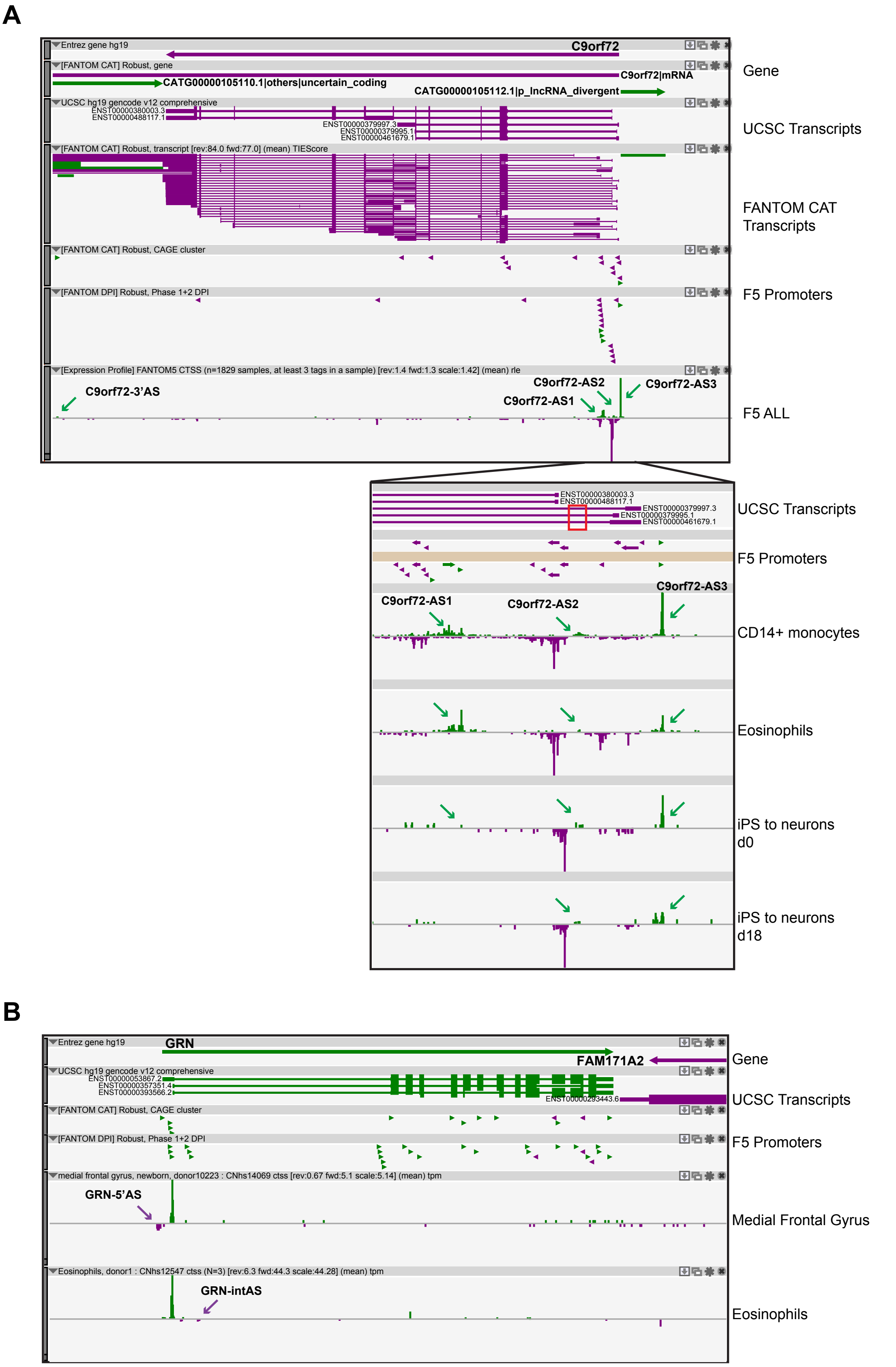

Supplementary Figure S3. Antisense transcription in FTD-associated genes. Zenbu Genome Browser view of *C9orf72* **(A)** and *GRN* **(B)** *loci*. Genes and transcripts are color-coded according to their orientation in the genome (+ strand, green; - strand, purple). Annotated genes and transcrips (refseq) are shown. FANTOM CAT assembled transcripts and FANTOM5 promoters are indicated according to their orientation in the genome. hCAGE data are presented for all FANTOM5 libraries pooled together (F5 ALL) or for selected samples, as indicated. Arrows highlight antisense TSSs. Exanucleotide repeat expansion in the *C9orf72* gene is shown (red box, A).

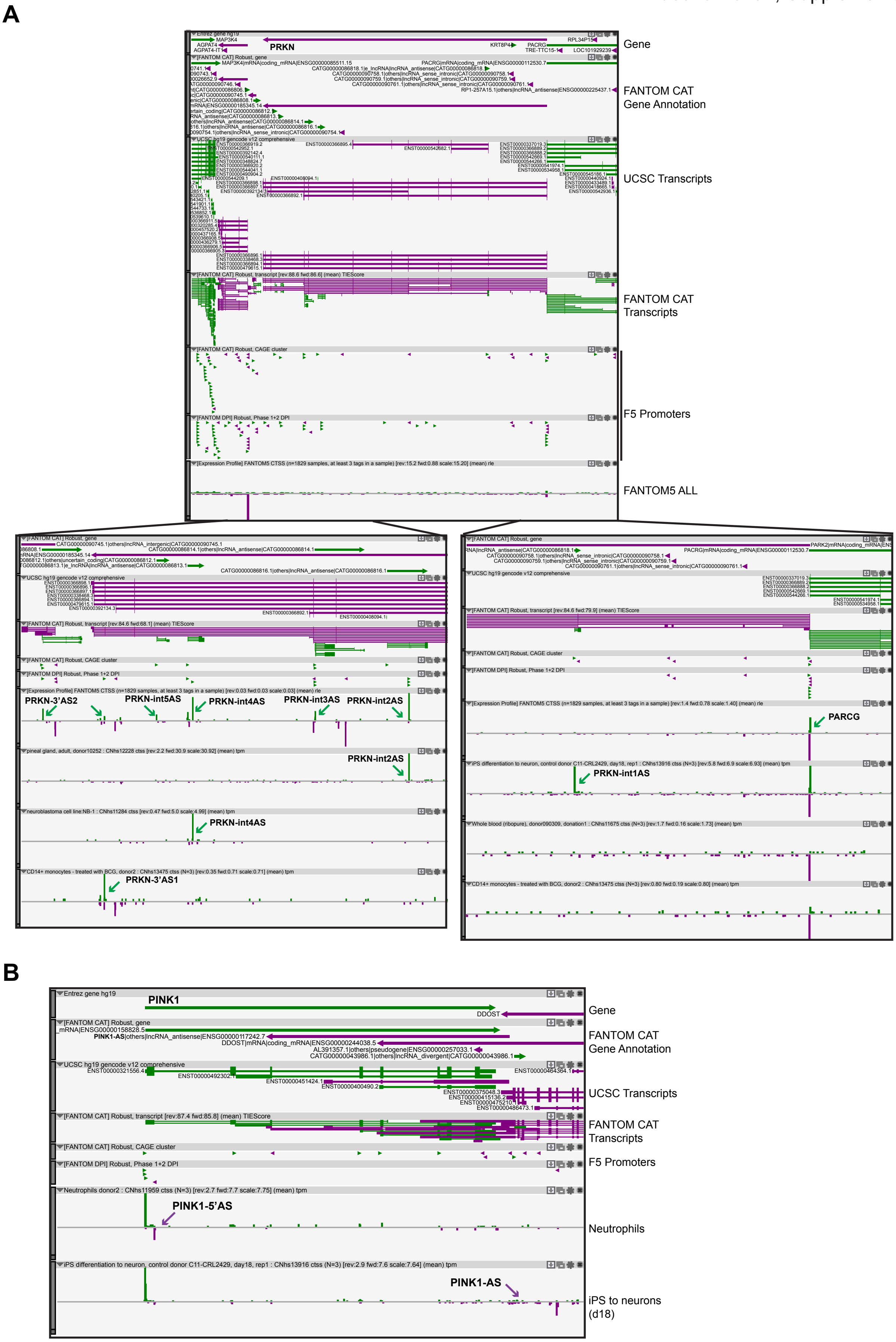

Supplementary Figure S4. Antisense transcription in genes associated to familial forms of PD. **A)** Zenbu Genome Browser view of *PRKN locus*. *PRKN* is expressed on the minus strand of the genome (purple signals). Main transcription initiation site for protein-coding Parkin co-regulated gene (*PARCG*) is also indicated. Green arrows highlight expression of additional intragenic AS transcripts. Zoom images are provided for the 5' (right) and 3' (left) regions of *PRKN*. **B)** Zenbu Genome Browser view of *PINK1 locus*. FANTOM gene and transcript assemblies are shown. Libraries with highest expression of PINK1 AS TSSs in brain and blood have been selected (neutrophils, iPSC differentiation into neurons). Purple arrow highlights previously published PINK1-AS (at the 3') and newly identified PINK1-5'AS (at the 5').

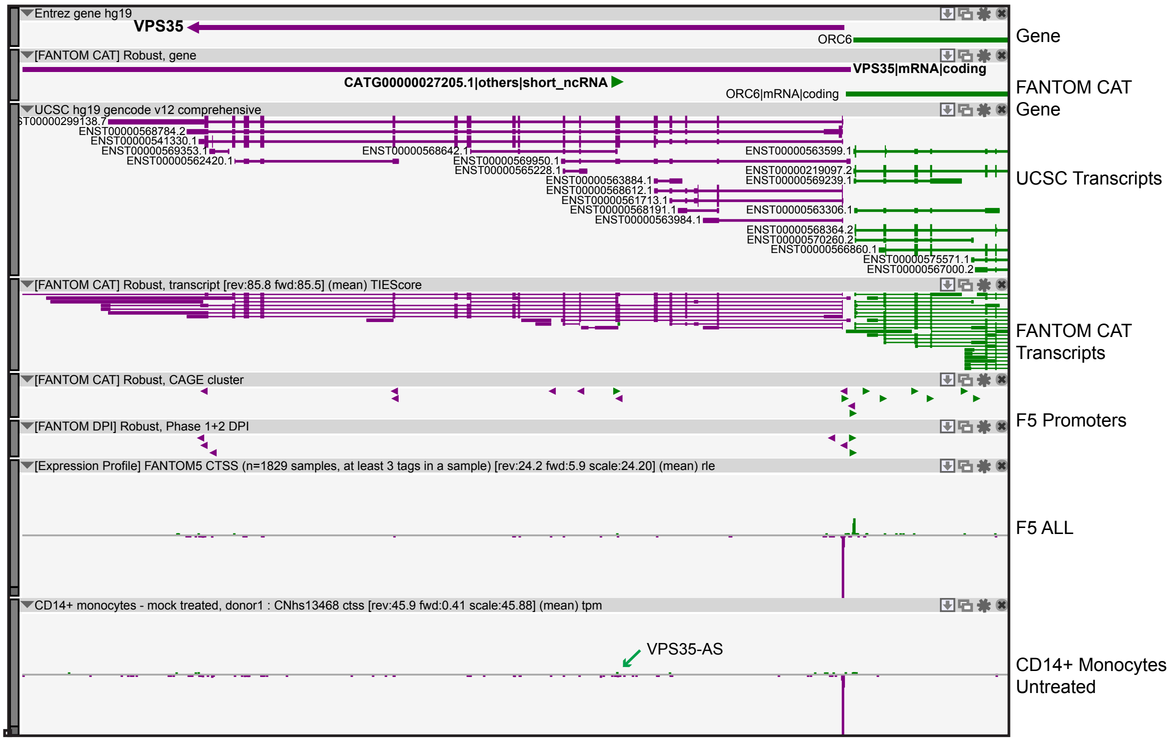

Supplementary Figure S5. Antisense transcription at *VPS35 locus*. Zenbu Genome Browser views of *VPS35* and *VPS35-AS* genes anatomy and expression. hCAGE data are shown for pooled FANTOM5 libraries (F5 ALL) and CD14^{+} monocytes.

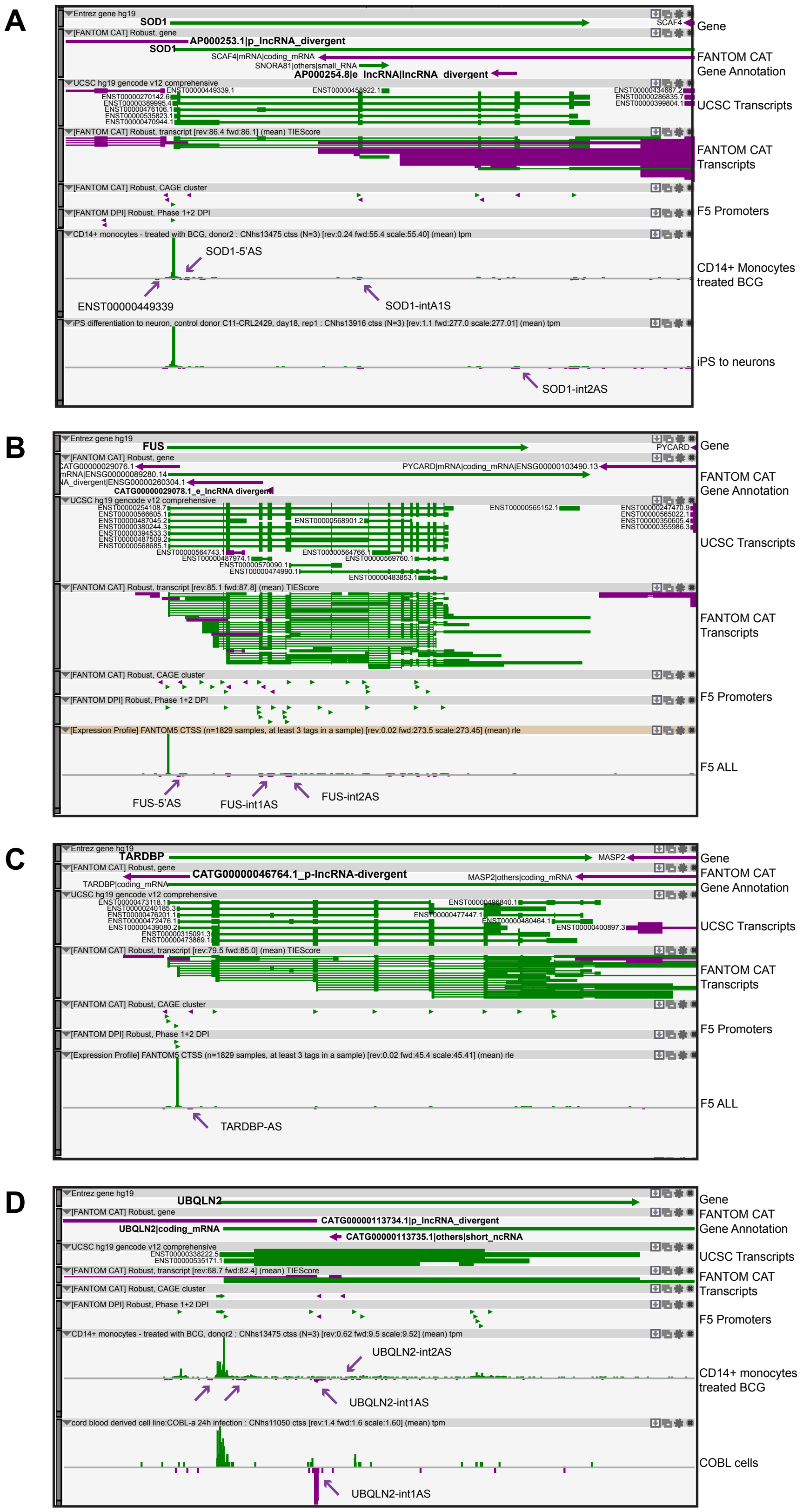

Supplementary Figure S6. Antisense transcription in genes associated to familial forms of ALS. Zenbu Genome Browser views of hCAGE data in selected brain and blood libraries indicate antisense transcription at genomic *loci* for *SOD1* **(A)**, *FUS* **(B)**, *TARDBP* **(C)** and *UBQLN2* **(D)**. Annotation of antisense lncRNAs from FANTOM CAT is highlighted.

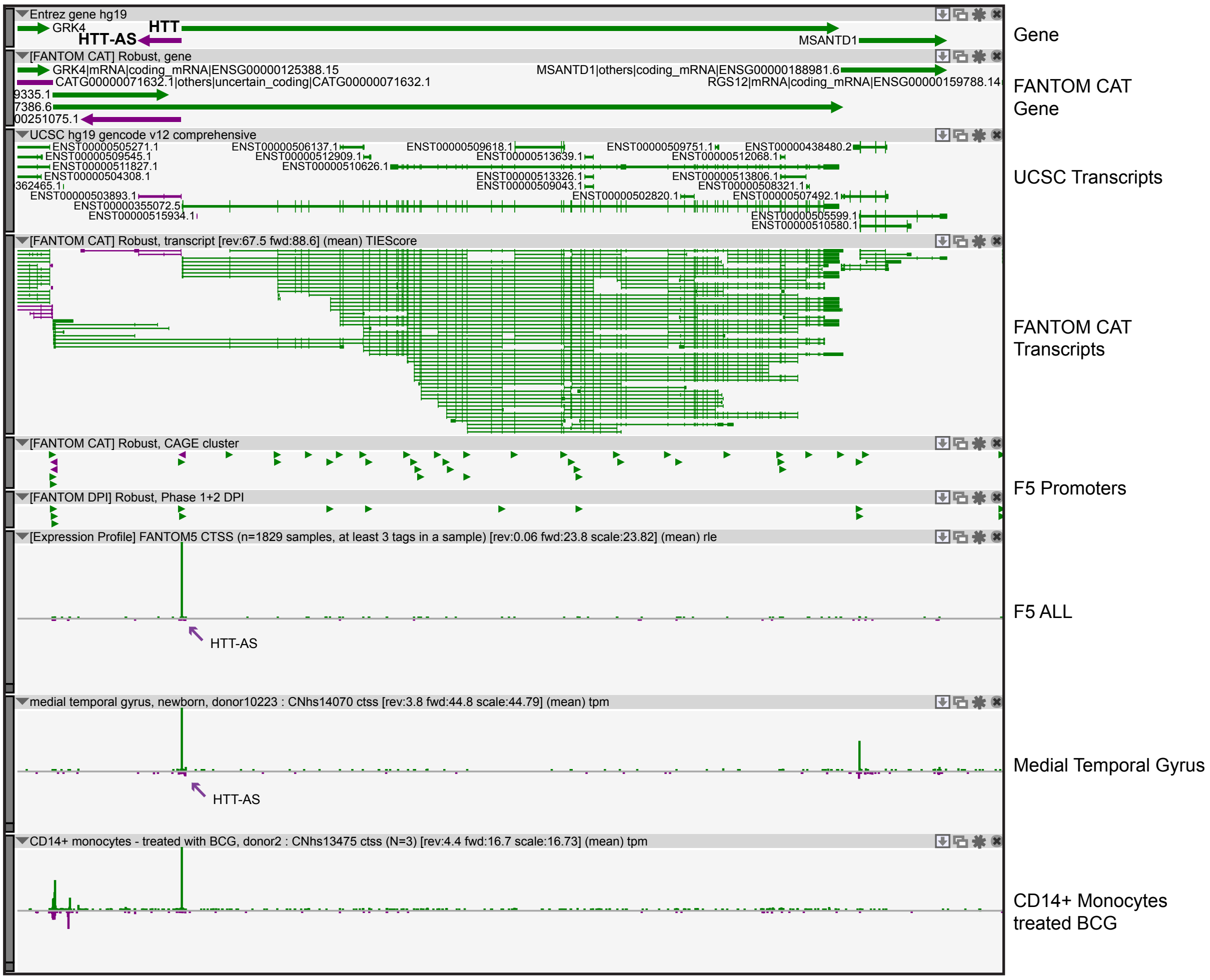

Supplementary Figure S7. Antisense transcription in the *HTT locus*. FANTOM CAT transcripts are shown, indicating the complexity of gene transcription in both orientations. Expression of *HTT* and *HTT-AS* is highlighted in pooled FANTOM5 libraries (F5 ALL), in Medial Temporal Gyrus and in CD14^{+} monocytes.
