# Supplementary material for: Antisense Transcription in Loci Associated to Hereditary Neurodegenerative Diseases
Source: Mol Neurobiol. 2019 Jan 4;56(8):5392–415. doi: 10.1007/s12035-018-1465-2 (PMC6614138; doi:10.1007/s12035-018-1465-2)
Supplement: Supplementary file 2 — (PDF 329 kb) [file 12035_2018_1465_MOESM2_ESM.pdf]

Antisense transcription in *loci* associated to hereditary neurodegenerative diseases

Silvia Zucchelli[1,2], Stefania Fedele[1], Paolo Vatta[1,3], Raffaella Calligaris[1,4], Peter Heutink[5,6,7,8], Patrizia Rizzu[5,9], Masayoshi Itoh[7,8,10], Francesca Persichetti[2], Claudio Santoro[2], Hideya Kawaji[7,8,10,11], Timo Lassmann[7,8,12,13], Yoshihide Hayashizaki[8,10], Piero Carninci[7,8,14], Alistair R.R. Forrest[7,8,15], the FANTOM Consortium and Stefano Gustincich[1,3]

	Gene Name	TSS DPI Permissive
AD	*APP*	48
AD	*PSEN1*	8
AD	*PSEN2*	1
FTD	*C9orf72*	14
FTD	*GRN*	39
FTD	*MAPT*	74
PD	*SNCA*	20
PD	*PRKN*	50
PD	*PINK1*	22
PD	*PARK7*	5
PD	*LRRK2*	23
PD	*VPS35*	33
ALS	*SOD1*	11
ALS	*FUS*	30
ALS	*TARDBP*	4
ALS	*UBQLN2*	10
HD	*HTT*	11
	Total	**403**

Supplementary Table S1. Catalogue of FANTOM5 permissive promoters (TSS DPI permissive) in *loci* associated to hereditary neurodegenerative diseases. The TOTAL number of permissive promoters identified in this study is also shown (grey box). AD, Alzheimer's disease; FTD, Frontotemporal Dementia; PD, Parkinson's disease; ALS, Amyotrophic Lateral Sclerosis; HD, Huntington's disease.

		FANTOM CAT Annotated TRAITS							
**Gene Name**	**AS lncRNA gene**	**Trait#1**	**Trait#2**	**Trait#3**	**Trait#4**	**Trait#5**	**Trait#6**	**Trait#7**	**Trait#8**
APP	*AP000230.1*	Insulin resistance	Type 2 diabetes	Schizophrenia					
APP	*CATG00000055739.1*	ND							
PSEN1	*CATG00000021544.1*	ND							
C9orf72	*CATG00000105112.1*	Amyotrphic Lateral Sclerosis	Prion Disease	Inflammation	Reumatoid Arthritis	Prostate Cancer	Heart disease	Abnormality of blood	Dementia
MAPT	*MAPT-AS1*	Parkinson Disease	Primary biliary cirrhosis	Alopecia	Sickle Cell Trait	Abnormality of RBC	Type 2 diabetes		
MAPT	*CATG00000033807.1*	Parkinson Disease	Primary biliary cirrhosis	Alopecia	Sickle Cell Trait	Abnormality of RBC	Type 2 diabetes	Lung Disease	Abnormality Lung
MAPT	*CATG00000033811.1*	Parkinson Disease	Primary biliary cirrhosis	Alopecia	Sickle Cell Trait	Abnormality of RBC	Type 2 diabetes		
MAPT	*CATG00000033813.1*	Parkinson Disease	Primary biliary cirrhosis	Alopecia	Sickle Cell Trait	Abnormality of RBC	Type 2 diabetes		
SNCA	*RP11-67M1.1 (SNCA-AS1)*	Parkinson Disease							
SNCA	*RP11-115D19.1*	Parkinson Disease	Prion Disease						
PRKN	*CATG00000086818.1*	ND							
PRKN	*CATG00000086816.1*	ND							
PRKN	*CATG00000086814.1*	Inherited metabolic disorder	Abnormality of metabolism	Auditory system disease	Hearing impairment				
PRKN	*CATG00000086813.1*	Atherosclerosis							
PINK1	*PINK1-AS*	ND							
LRRK2	*AC079630.4*	Parkinson Disease	Crohn's disease	Asthma	Sclerosis upper limbs	Glomerulosclerosis	Multiple Sclerosis	Arteriosclerosis	
SOD1	*AP000253.1*	Esophageal Cancer	Sickle Cell Anemia						
SOD1	*AP000254.8*	ND							
FUS	*CATG00000029076.1*	ND							
FUS	*RP11-388M20.6*	Blood coagulation	Atrial fibrillation	Pulmonary embolism	Abnormality of acid base	Pulmonary systemic sclerosis	IgA glomerulonephritis	Graves disease	Systemic lupus erythematosus
FUS	*CATG00000029078.1*	Blood coagulation	Atrial fibrillation	Pulmonary embolism	Abnormality of acid base	Pulmonary systemic sclerosis	IgA glomerulonephritis	Graves disease	Systemic lupus erythematosus
TARDBP	*CATG00000046764.1*	ND							
UBQLN2	*CATG00000113734*	ND							
HTT	*HTT-AS*	Abnormality of the myocardium							

Supplementary Table S2. List of FANTOM CAT GWAS traits associated to antisense lncRNAs in *loci* associated to hereditary neurodegenerative diseases.

Disease	Gene Name	AS lncRNA gene	Ontology Name	Ontology ID	Fold Change	p-Value
AD	*APP*	*AP000230.1*	nervous system	UBERON:0001016	24.7	3.71E-38
			brain	UBERON:0000955	24.2	5.20E-38
AD	*APP*	*CATG00000055739.1*	heart blood vessel	UBERON:0003498	5.57	0.0111
			smooth muscle cell (artery)	CL:0002592	7.62	0.0077
AD	*PSEN1*	*CATG00000021544.1*	immune system	UBERON:0002405	5.05	9.65E-25
			hematopoietic system	UBERON:0002390	6.52	4.35E-36
			hematopoietic cell	CL:0000988	51.4	1.00E-31
FTD	*C9orf72*	*CATG00000105112.1*	hematopoietic system	UBERON:0002390	12	5.65E-31
			immune system	UBERON:0002405	9.94	1.81E-18
			leukocyte	CL:0000738	35.5	7.59E-50
			hematopoietic cell	CL:0000988	33.8	2.68E-46
FTD	*MAPT*	*MAPT-AS1*	nervous system	UBERON:0001016	44.1	1.00E-44
			brain	UBERON:0000955	32.7	6.73E-41
			neuron	CL:0000540	5.07	1.11E-02
FTD	*MAPT*	*CATG00000033807.1*	ND	ND	ND	ND
FTD	*MAPT*	*CATG00000033811.1*	parietal lobe	UBERON:0001872	8.76	0.0192
FTD	*MAPT*	*CATG00000033813.1*	nervous system	UBERON:0001016	67.1	2.49E-23
			brain	UBERON:0000955	34.7	4.33E-21
			neuron	CL:0000540	15.7	0.00343
PD	*SNCA*	*RP11-67M1.1 (SNCA-AS1)*	blood	UBERON:0000178	187	2.46E-07
			reticulocyte	CL:0000558	9.41	0.0171
PD	*SNCA*	*RP11-115D19.1*	placenta	UBERON:0001987	10.9	0.0016
			mesothelial cells	CL:0000077	19.7	0.00371
			endothelial cells	CL:0000066	5.22	2.85E-21
PD	*PRKN*	*CATG00000086818.1*	extra-ocular muscle	UBERON:0001601	45.1	0.0113
			medulla oblungata	UBERON:0001896	35	0.000913
			spinal cord	UBERON:0002240	10.1	0.0307
			synovial cells	CL:0000214	11.5	0.00471
PD	*PRKN*	*CATG00000086816.1*	ND	ND	ND	ND
PD	*PRKN*	*CATG00000086814.1*	pituitary gland	UBERON:0000007	47	0.00312
PD	*PRKN*	*CATG00000086813.1*	adipose tissue	UBERON:0001013	6.95	0.000506
			spinal cord	UBERON:0002240	5.88	0.00889
			fat cells	CL:0000136	15.1	0.00000153
PD	*PINK1*	*PINK1-AS*	ND	ND	ND	ND
PD	*LRRK2*	*AC079630.4*	hematopoietic system	UBERON:0002390	5.92	2.92E-25
			leukocyte	CL:0000738	9.02	1.09E-14
			hematopoietic cell	CL:0000988	8.52	4.71E-13
			blood	UBERON:0000178	10.3	7.74E-07
ALS	*SOD1*	*AP000253.1*	testis	UBERON:0000473	15.5	0.0146
ALS	*SOD1*	*AP000254.8*	hepatocytes	CL:0000182	5.93	0.0168
ALS	*FUS*	*CATG00000029076.1*	testis	UBERON:0000473	12.2	0.0179
			B cells	CL:0000236	10.9	8.91E-07
ALS	*FUS*	*RP11-388M20.6*	ND	ND	ND	ND
ALS	*FUS*	*CATG00000029078.1*	ND	ND	ND	ND
ALS	*TARDBP*	*CATG00000046764.1*	ND	ND	ND	ND
ALS	*UBQLN2*	*CATG00000113734*	pineal body	UBERON:0001905	12.4	0.0091
			NK cells	CL:0000623	7.89	0.00422
HD	*HTT*	*HTT-AS*	pineal body	UBERON:0001905	18.8	0.00429
			nervous system	UBERON:0001016	16.9	1.54E-16
			brain	UBERON:0000955	15.2	7.76E-17

Supplementary Table S3. Sample Ontology Enrichment. List of the most enriched sample ontology categories (Ontology name, ontology ID) associated to FANTOM CAT antisense lncRNAs in the *loci* associated to hereditary neurodegenerative diseases analyzed in this study. Fold change and p-values indicate enrichment in the specific term versus all the other samples.

Disease	Gene Symbol	AS TSS	Anatomy	Reference	FANTOM CAT classification
AD	*APP*	APP-bAS	5' head-to-head		
AD	*APP*	APP-5'AS	5' head-to-head	FANTOM CAT	p-lncRNA_divergent
AD	*APP*	APP-int1AS	5' head-to-head		
AD	*APP*	APP-int2AS	intragenic	FANTOM CAT	others_short ncRNA
AD	*APP*	APP-int3AS	intragenic	FANTOM CAT	others_lncRNA_divergent
AD	*APP*	APP-3'AS	3' tail-to-tail	FANTOM CAT	others_short ncRNA
AD	*PSEN1*	PSEN1-5'AS	5' head-to-head	FANTOM CAT	p_lncRNA_divergent
AD	*PSEN1*	PSEN1-bAS	bidirectional		
AD	*PSEN1*	PSEN1-intAS	intragenic	FANTOM CAT	others_short ncRNA
AD	*PSEN2*	PSEN2-5'AS	5' head-to-head		
AD	*PSEN2*	PSEN2-intAS	intragenic		
FTD	*C9orf72*	C9orf72-AS3	bidirectional	FANTOM CAT; Rizzu P., et al., 2016	p_lncRNA_divergent
FTD	*C9orf72*	C9orf72-AS2	5' head-to-head	Rizzu P., et al., 2016	
FTD	*C9orf72*	C9orf72-AS1	5' head-to-head	Rizzu P., et al., 2016; Zu T., et al., 2013	
FTD	*C9orf72*	C9orf72-3'AS	3' tail-to-tail		
FTD	*GRN*	GRN-5'AS	bidirectional		
FTD	*GRN*	GRN-intAS	5' head-to-head		
FTD	*MAPT*	MAPT-AS1	5' head-to-head	FANTOM CAT	p_lncRNA_divergent
FTD	*MAPT*	MAPT-int1AS	intragenic	FANTOM CAT	others_lncRNA_antisense
FTD	*MAPT*	MAPT-int2AS	intragenic	FANTOM CAT	others_lncRNA_antisense
FTD	*MAPT*	MAPT-int3AS	intragenic	FANTOM CAT	e_lncRNA_divergent
FTD	*MAPT*	MAPT-3'AS	3' tail-to-tail		
PD	*SNCA*	SNCA-5'AS	5' head-to-head		
PD	*SNCA*	ENST00000501215	5' head-to-head	Annotated lncRNA (ENSEMBL)	
PD	*SNCA*	SNCA-int1AS	5' head-to-head	FANTOM CAT	others_lncRNA_divergent
PD	*SNCA*	SNCA-int2AS	intragenic	FANTOM CAT	others_short ncRNA
PD	*SNCA*	SNCA-3'AS1	3' tail-to-tail		
PD	*SNCA*	SNCA-3'AS2	3' tail-to-tail	FANTOM CAT	e_lncRNA_intergenic
PD	*SNCA*	ENST00000513572	3' tail-to-tail	Annotated lncRNA (ENSEMBL)	
PD	*PRKN*	PARCG	5' head-to-head	West A.B., et al., Jmol Biol, 2003	mRNA_prot coding
PD	*PRKN*	Parkin-int1AS	intragenic	FANTOM CAT	e-lncRNA_antisense
PD	*PRKN*	Parkin-int2AS	intragenic	FANTOM CAT	others_lncRNA_antisense
PD	*PRKN*	Parkin-int3AS	intragenic	FANTOM CAT	others_lncRNA_antisense
PD	*PRKN*	Parkin-int4AS	intragenic	FANTOM CAT	e-lncRNA_antisense
PD	*PRKN*	Parkin-int5AS	intragenic	FANTOM CAT	others_uncertain_coding
PD	*PRKN*	Parkin-3'AS1	3' tail-to-tail		
PD	*PRKN*	Parkin-3'AS2	intergenic	FANTOM CAT	e-lncRNA_intergenic
PD	*PINK1*	PINK1-5'AS	5' head-to-head		
PD	*PINK1*	PINK1-AS	3' tail-to-tail	Scheele C., et al., BMC Genomics, 2007	others_lncRNA_antisense
PD	*PARK7*	DJ1-5'AS	bidirectional		
PD	*PARK7*	DJ1-intAS	5' head-to-head		
PD	*LRRK2*	LRRK2-bAS	bidirectional		
PD	*LRRK2*	LRRK2-5'AS	5' head-to-head	FANTOM CAT	p_lncRNA_divergent
PD	*LRRK2*	LRRK2-int1AS	5' head-to-head		
PD	*LRRK2*	LRRK2-int2AS	5' head-to-head		
PD	*VPS35*	VPS35-AS	intragenic	FANTOM CAT	others_short ncRNA
ALS	*SOD1*	ENST00000449339	bidirectional	Annotated lncRNA	
ALS	*SOD1*	SOD1-5'AS	5' head-to-head	FANTOM CAT	p_lncRNA_divergent
ALS	*SOD1*	SOD1-int1AS	intragenic		
ALS	*SOD1*	SOD1-int2AS	intragenic	FANTOM CAT	e_lncRNA_divergent
ALS	*FUS*	FUS-5'AS	5' head-to-head	FANTOM CAT	p_lncRNA_divergent
ALS	*FUS*	FUS-int1AS	intragenic	FANTOM CAT	e_lncRNA_divergent
ALS	*FUS*	FUS-int2AS	intragenic	FANTOM CAT	e_lncRNA_divergent
ALS	*TARDBP*	TARDBP-AS	5' head-to-head	FANTOM CAT	p_lncRNA_divergent
ALS	*UBQLN2*	UBQLN2-bAS	bidirectional		
ALS	*UBQLN2*	UBQLN2-5'AS	5' head-to-head		
ALS	*UBQLN2*	UBQLN2-intAS	intragenic	FANTOM CAT	p_lncRNA_divergent
HD	*HTT*	HTT-AS	5' head-to-head	Chung D.W., et al., HMG, 2011	p_lncRNA_divergent

Supplementary Table S4. List of natural antisense transcripts to neurodegeneration-associated genes identified in this study. Identified transcripts are listed in alphabetical order based on the disease and gene name (Gene Symbol), as discussed in the manuscript. AS TSS nomenclature and genomic anatomy are indicated. References to FANTOM CAT catalogue of human lncRNAs and classification of lncRNA functions are included. Reference to published work is shown, when available.
